# FG-3019, a Human Monoclonal Antibody Recognizing Connective Tissue Growth Factor, is Subject to Target-Mediated Drug Disposition

**DOI:** 10.1007/s11095-016-1918-0

**Published:** 2016-04-08

**Authors:** Mitchell C. Brenner, Wojciech Krzyzanski, James Z. Chou, Pierre E. Signore, Cyra K. Fung, David Guzman, Dongxia Li, Weihua Zhang, David R. Olsen, Viet-Tam L. Nguyen, Carolyn W. Koo, Mark D. Sternlicht, Kenneth E. Lipson

**Affiliations:** FibroGen, Inc., 409 Illinois St., San Francisco, California 94158 USA; Department of Pharmaceutical Sciences, State University of New York at Buffalo, 370 Kapoor Hall, Buffalo, New York 14214 USA

**Keywords:** Connective Tissue Growth Factor, CTGF, FG-3019, TMDD

## Abstract

**Purpose:**

To evaluate and model the pharmacokinetic and pharmacodynamic behavior in rats of FG-3019, a human monoclonal antibody targeting connective tissue growth factor (CTGF).

**Methods:**

FG-3019, human CTGF (rhCTGF), or the N-terminal domain of rhCTGF were administered intravenously to rats and concentrations of these proteins as well as endogenous CTGF were determined by immunoassays. FG-3019, or ^125^I-labeled FG-3019, and human CTGF (rhCTGF) were co-administered to assess the impact of CTGF on the elimination rate and tissue localization of FG-3019, which was further characterized by immunohistochemical analysis. A PK/PD model for target-mediated elimination of FG-3019 was developed to fit the kinetic data.

**Results:**

FG-3019 exhibited non-linear pharmacokinetics in rats. Circulating concentrations of the N-terminal half of CTGF increased after dosing with FG-3019, reached maximal levels after 1–5 days, and returned toward baseline levels as FG-3019 cleared from the circulation, whereas the concentration of intact CTGF was unaffected by administration of FG-3019. Co-administration of rhCTGF dramatically enhanced the rate of FG-3019 elimination, redistributing the majority of ^125^I-labeled FG-3019 from the blood to the liver, kidney, spleen and adrenal gland. FG-3019 co-administered with CTGF was found along the sinusoids of the liver and adrenal glands, the capillaries of the kidney glomeruli and in the spleen. A pharmacokinetic model for target-mediated elimination of FG-3019 was used to fit the time courses of FG-3019 and endogenous CTGF plasma concentrations, as well as time courses of rhCTGF and rhCTGF N-fragment after intravenous administration of these species.

**Conclusions:**

FG-3019 is subject to target mediated elimination in rats.

**Electronic supplementary material:**

The online version of this article (doi:10.1007/s11095-016-1918-0) contains supplementary material, which is available to authorized users.

## Introduction

Connective tissue growth factor (CTGF, CCN2) is a member of a small family of secreted monomeric proteins that are characterized by their highly conserved disulfide bonding pattern and organization into 3–4 domains having homology to other proteins (1). The four domains of CTGF are homologous to 1) IGF-1 binding proteins, 2) the von Willebrand factor type C repeat, 3) the thrombospondin type 1 repeat, and 4) a cysteine knot motif common to proteins that bind heparan sulfate proteoglycans (HSPGs), respectively. A protease susceptible linker (hinge region) interconnects domains 2 and 3, which upon cleavage produces CTGF-N (domains 1–2) and CTGF-C (domains 3–4). CTGF-N appears to be proteolytically stable, as it is the portion of CTGF that is commonly observed in biological fluids like plasma or urine, whereas CTGF or CTGF-C is usually not observed in biological fluids at appreciable concentrations (2).

Increased concentrations of CTGF have been reported in various chronic diseases including liver fibrosis, systemic sclerosis, diabetic nephropathy, as well as pancreatic cancer (3). Because of the association between elevated CTGF concentrations and progression of tissue remodeling diseases, inhibition of CTGF has been suggested as a therapeutic target (4). FG-3019 is a human anti-CTGF IgG1 monoclonal antibody currently under clinical investigation as a potential therapeutic for treatment of idiopathic pulmonary fibrosis, liver fibrosis and pancreatic cancer (5). FG-3019 was selected for clinical development from a panel of anti-CTGF antibodies based on its ability to recognize both human and rodent CTGF and its activity in functional assays (6). As part of the selection process, FG-3019 and several other anti-CTGF antibodies were evaluated for pharmacokinetic performance in rats. Antibodies specific to human CTGF exhibited slower clearance and longer half-lives in rats than antibodies that recognized rat as well as human CTGF (unpublished observations), suggesting the potential for target-mediated antibody elimination. Here we report the assessment of FG-3019 PK in rats following IV administration, which is characterized by relatively rapid dose-dependent clearance and dose-dependent terminal half-life. We present additional experiments to understand the observed non-linear kinetics in terms of a target-mediated clearance mechanism. These experiments include an assessment of the effect of exogenous CTGF on the elimination kinetics of FG-3019 in rats, the tissue distribution of radioiodinated FG-3019 in the absence and presence of exogenous CTGF, immunohistochemical localization of unlabeled FG-3019 in rat tissues, as well as kinetic modeling of target-mediated antibody elimination. Together these studies show that complexes formed between FG-3019 and CTGF *in vivo* are subject to extremely rapid elimination, which dominates the pharmacokinetics at low doses, but is a minor contributor to antibody elimination at high doses.

## Materials and Methods

### FG-3019

FG 3019 is a human, recombinant DNA-derived, IgG1κ monoclonal antibody that binds to CTGF in domain 2, with high affinity (K_d_ = 0.1–0.2 nM).

### Production of CTGF

Recombinant human and rat CTGF (CTGF-whole or CTGF-W) and CTGF-N were expressed in CHO cells as secreted proteins. The proteins were purified from concentrated conditioned media using an antibody affinity-based purification with FG-3019-Sepharose resin followed by ion exchange chromatography with SP-Sepharose Fast Flow resin (GE Healthcare).

### CTGF Antibodies

Human IgG1 monoclonal antibodies expressed in CHO cells that target the N- and C-half portions of CTGF, respectively, were used to assay for CTGF forms having an intact hinge region connecting the two halves of CTGF. The N-half-reactive antibody binds to Domain 1 of CTGF and is referred to as mAb-D1. The C-half reactive antibody binds to Domain 3 of CTGF and is referred to as mAb-D3. We employed a bivalent Fab “mini” antibody targeting the N-half of CTGF (referred to as “minibody”) that contains a dHLX dimerization domain followed by a Myc-His peptide tag at the C-terminus of the antibody heavy chain. The minibody was expressed in *E.coli* and purified by immobilized metal ion chromatography (custom prepared for FibroGen by AbD Serotec (Puchheim, Germany)).

### Production of Receptor Associated Protein (RAP)

Receptor associated protein (Cat. No. IRAP-514) was prepared by Innovative Research, Inc. (Novi, MI). The protein was expressed as a glutathione-S-transferase fusion protein in *E.coli* and purified by glutathione-agarose chromatography. The fusion protein was cleaved with thrombin and GST-containing species were removed by glutathione-agarose chromatography. RAP was further purified by heparin sepharose affinity chromatography (7). The purified protein (0.81 mg/ml based on ϵ_280_ = 0.93) was dialyzed against 20 mM ammonium bicarbonate and then lyophilized. The final purified material was judged greater than 95% pure by SDS-PAGE. The N-terminus of the final product has sequence GSYSREKN, which differs from the native N-terminus of YSREKN due to the remnant sequence (Gly –Ser) from the thrombin cleavage consensus sequence.

### Pharmacokinetics of FG-3019 in Rats

Male Sprague–Dawley rats (purchased pre-cannulated from Charles River Labs, Hollister, CA) weighing an average of 336 g were housed, two per cage, with free access to food and water throughout the study. FG-3019 formulated at 0.01, 0.1, 1, 3 and 10 mg/ml was sterile filtered and administered (3 ml/kg or 10 ml/kg for the highest FG-3019 dose) to anesthetized rats through tail vein injection at 0.03, 0.3, 3, 10, 30 and 100 mg/kg. Blood samples (0.25 ml) were collected at one pre-dose and 12 post-dose time points into lithium heparin coated tubes from a catheter implanted in the jugular vein. Plasma was isolated by centrifugation and stored at −80°C. Results from multiple experiments were combined for pharmacokinetic analysis.

### Pharmacokinetics of Recombinant Human CTGF in Rats

Procedures were similar to those for assessment of the pharmacokinetics of FG-3019. Recombinant human CTGF (0.253 and 0.523 mg/ml) and CTGF-N (0.127 and 0.267 mg/ml) were sterile filtered in 50 mM Tris–HCl, 800 mM sodium chloride, pH 7.5, and administered (3 ml/kg) to obtain four groups of animals (3 rats/group) dosed at 0.76 and 1.6 mg/kg with CTGF and 0.38 and 0.80 mg/kg with CTGF-N (20 and 40 nmol/kg of each form of CTGF). Blood sample collection procedures were the same as for FG-3019.

### Measurement of FG-3019 in Rat Plasma

FG-3019 was measured by capturing with an *E.coli* produced CTGF Exon-3 peptide and detecting with a goat anti-human kappa antibody conjugated with horseradish peroxidase (Cat. No. 2060–05, Southern Biotech, AL). All samples underwent a minimum dilution of 10-fold in buffer, with further dilutions made in 10% pooled lithium heparin rat plasma. Calibrators were prepared by spiking recombinant FG-3019 into 10% pooled rat plasma.

In samples containing high concentrations of recombinant human CTGF and in samples from low-dose (0.03 – 3 mg/kg FG-3019) PK experiments, FG-3019 was measured using either a human IgG assay kit purchased from Cygnus Technologies (Cat. No. F160), or by a similar assay in which FG-3019 was captured with an anti-human IgG (Fc specific) antibody (Cat. No.12136, Sigma) and detected using a goat anti-human kappa antibody conjugated with horseradish peroxidase (Cat. No. 2060–05, Southern Biotech, AL). All samples underwent a minimum dilution of 5-fold in buffer, with further dilutions made in sample diluent provided by the vendor (for samples measured using the Cygnus Technologies assay kit) or in 20% pooled lithium heparin rat plasma. Calibrators were prepared following the vendor’s instructions (Cygnus Technologies assay kit) or by spiking recombinant FG-3019 into 20% pooled rat plasma.

Final values for each sample were determined based on the average of results for multiple dilutions having values within 20% of each other. These assays were confirmed not to be subject to interference by human CTGF.

### Assays for Rat CTGF

CTGF was measured in rat plasma using one of two immunoassays (see Figure S1). One assay employed capture with mAb-D3 and detection with a Domain 2 reactive minibody conjugated with alkaline phosphatase. This assay detects forms of CTGF that have an intact hinge region connecting Domains 2 and 3, and is referred to as a W-CTGF assay. Calibrators were prepared by spiking recombinant rat CTGF into pooled rat plasma. The second assay employed capture with the Domain 2 reactive minibody followed by detection with FG-3019 and a goat anti-human kappa antibody conjugated with horseradish peroxidase (Cat. No. 2060–05, Southern Biotech, AL). Calibrators were prepared by spiking recombinant rat CTGF-N into pooled rat plasma. This assay (the N+W-CTGF assay) detects forms of CTGF containing Domain 2, which includes the N-terminal half of CTGF and full length CTGF. Neither of the two CTGF assays is subject to interference by FG-3019.

### Assay for rhCTGF in Rat Plasma

Plasma from rats dosed with recombinant human CTGF were assayed for CTGF using two ELISAs specific to human CTGF (see Figure S1). To measure CTGF, standards, samples and quality control samples were added to 96-well plates coated with 5 μg/ml mAb-D3. After washing the plates, human CTGF was detected by incubation for 1.5 h with 500 ng/ml alkaline phosphatase-labeled mAb-D1 followed by color formation generated after addition of pNPP reagent. mAb-D1 does not bind to rodent forms of CTGF, making this W-CTGF assay selective for human CTGF. A second CTGF assay (human N+W-CTGF) employed microtiter plates coated with the minibody which binds to Domain 2 of human (and rat) CTGF. Human CTGF was then detected using the human specific alkaline phosphatase-labeled mAb-D1. This N+W-CTGF assay detects both intact rhCTGF and the N-terminal fragment of rhCTGF. Calibrator data for each of the ELISA methods described above were fit to 4-parameter logistic equations.

### FG-3019 Pharmacokinetics in Rats Following Injection of CTGF or CTGF-N

Six rats per group were injected through the tail vein with FG-3019 (3 mg/kg, dose volume 3 ml/kg). Approximately 10 min after dosing with FG-3019 (Figure S2), animals were dosed with buffer, or equimolar amounts of CTGF or CTGF-N (3 ml/kg). Blood samples (0.2 ml) were collected pre-study, 5 min before injection of CTGF, and then 5 min, 15 min, 0.5 h, 1 h, 3 h, 7 h, 24 h, 48 h, 3, 5, and 8 days after injection of CTGF. Each group was divided into two sub-groups of three animals, with blood sampling conducted at alternating time points so that no animals had more than four blood samplings over a 24-h period. A 0.25 ml priming blood sample was collected prior to the 0.20 ml blood samples. The 0.25 ml priming sample was re-injected into the animal together with 0.2 ml of 10 U/ml heparinized saline after each blood sample collection. Blood was centrifuged at 3510×*g* for 10 min and the plasma collected and stored at −80°C.

### FG-3019 Pharmacokinetics in Rats Co-Dosed with Different Amounts of CTGF

Three rats per group were injected through the tail vein with FG-3019 (3 mg/kg, dose volume 3 ml/kg). Approximately 10 min after dosing with FG-3019 (Figure S3), rats were injected through the tail vein with bolus doses of CTGF (or buffer), at molar ratios of CTGF:FG-3019 equal to 0:1, 0.5:1, 1:1 and 2:1 (3 ml/kg). Blood samples (0.20 ml) were collected into lithium heparin coated tubes pre-study, 5 min before the dose of CTGF, and at 3 min, 6 min, 0.5, 6, 24, 48 and 144 h after the dose of CTGF. Blood samples (0.2 ml) were collected and processed as described above.

### FG-3019 Pharmacokinetics in Rats Co-administered with RAP and CTGF

Rats were cannulated through the right jugular (for blood collection) and left femoral veins (for dose administration) and allowed to recover at least 48-h after surgery before initiation of dosing. FG-3019 was administered to conscious animals (*n* = 3 or 4 per group; 1.5 ml/kg) (Figure S4), followed 10 min later by RAP (or vehicle; 0.9 ml), which was followed immediately by CTGF (or vehicle; 1.5 ml/kg). Catheters were flushed with 0.15 ml of sterile saline after each administration of each test article. Blood samples (0.2 ml) were collected and processed as described above.

### Preparation of [^125^I]-FG-3019

[^125^I]-FG-3019 (~2 mCi/mg) was prepared by Vitrax Radiochemicals (Placentia, CA) by chemical radioiodination of FG-3019. The radioiodinated antibody was checked for its ability to bind CTGF by comparison with non-radioiodinated FG-3019 in a direct ELISA based on solid phase capture by CTGF Exon-3 peptide, which corresponds to Domain 2 of CTGF. The radioiodinated and non-iodinated antibodies yielded comparable dilutional behavior in the ELISA. The [^125^I]-FG-3019 was diluted to 2 mCi/mg with non-radioiodinated FG-3019 in 0.9% saline prior to injection.

### Tissue Distribution and Excretion Analysis of [^125^I]-FG-3019 in Rats

The following procedures were conducted at QPS, LLC (Newark, DE). Five rats were placed on study and were given water containing NaI (10 mg/ml) beginning at least 48 h before test article dosing and during the study. Body weights of each animal were determined prior to and on the day of dosing. The radioactive concentration and homogeneity of the dose formulation was determined before and after dosing using gamma counting analysis. Each animal received a single tail vein injection of the formulation, which contained [^125^I]-FG-3019 in a vehicle of 0.9% saline to achieve the target dose of 10 mg/kg and a radioactivity dose of 20 μCi/kg at a dose volume of 5 ml/kg. One animal each was assigned to be euthanized at 0.25, 2, 6, and 24 h post-dose. One additional animal, which was given an IV dose of 5 mg/kg of rhCTGF approximately 10 min after the administration of [^125^I]-FG-3019, was euthanized at 0.25 h after the dose of [^125^I]-FG-3019 (5 min after administration of rhCTGF). At the scheduled times, animals were deeply anesthetized via isoflurane inhalation, a blood sample was obtained via cardiac puncture, and the animals were euthanized by freezing in a hexane dry-ice bath for quantitative whole body autoradiographic (QWBA) analysis. The animals were not perfused prior to freezing. Whole blood, plasma (K_2_EDTA anticoagulant), urine, feces, cage wash and wipes as well as carcasses were collected and stored at −70°C.

After removing the pinna, distal limbs, hair and tail, the frozen carcasses were embedded in 2% (*w*/*v*) carboxymethylcellulose and frozen into a block prior to sectioning. Internal quality control and calibration standards (blood fortified with [^125^I] sodium iodide) were placed into the frozen blocks prior to sectioning to control for section thickness and image calibration. Several sections approximately 40 μm thick were taken in the sagittal plane using a cryostat microtome set to −20°C. Sections were dehydrated prior to exposure to phosphorimaging screens. The exposed screens were scanned using a Molecular Dynamics Typhoon 9410 Phosphor Imager and data acquired as counts per mm^2^. Tissue concentrations of radioactivity were determined by interpolation from a standard curve based on the calibration standards and expressed in terms of μCi/gram, which was converted to microgram equivalents of [^125^I]-FG-3019 per gram of tissue based on the specific activity of [^125^I] -FG-3019. A single, best representative section was used from each animal for determination of tissue concentrations of radioactivity.

### Immunohistochemical Localization of FG-3019 and CTGF

Rats received a 3 mg/kg tail vein injection of FG-3019 followed 10 min later by a 1.52 mg/kg injection of rhCTGF (CTGF:FG-3019 molar ratio of 2:1) or buffer. Control animals were dosed with the FG-3019-vehicle followed 10 min later by buffer. Animals (*n* = 2 per group) were harvested 5 min after CTGF (or buffer) administration. The lungs were perfused with ice cold saline from the right ventricle for approximately 1 min. The animals were then perfused from the left ventricle with ice cold saline for 3 min followed by 10% buffered formalin for 3 min. The following tissues were harvested and stored in 10% buffered formalin: one liver lobe, both kidneys, both adrenal glands, spleen, cardiac ventricles and lungs. The lungs were stored inflated with 10% buffered formalin. Immunohistochemistry was performed on formalin-fixed, paraffin-embedded tissue sections. For antigen retrieval, specimens were boiled in TRIS/EDTA buffer (10 mM Tris Base, 1 mM EDTA Solution, 0.05% Tween 20, pH 9.0) for 20 min. Between incubations, slides were washed three times in TBST-buffer (Teknova, Hollister, CA). A specific anti-human CTGF mouse monoclonal antibody produced at FibroGen was used for CTGF detection and a rabbit anti-human IgG (Jackson Immunoresearch Lab, West Grove, PA) was used to detect FG-3019. A tyramide signal amplification system (TSA-kit, Perkin Elmer) was used for CTGF immunostaining and EnVision + system (DAKO, Carpinteria, CA) was used for FG-3019 immunostaining according to the manufacturer’s instructions. Peroxidase-diaminobenzidine was used as chromogen. Slides were cover-slipped with an aqueous mounting media (Cytoseal XYL, VWR, Visalia, CA).

### Non-Compartmental Pharmacokinetic Analysis

The PK parameters were estimated by a non-compartmental pharmacokinetic analysis using Phoenix WinNonlin™ 6.2 (Pharsight, A Certara™ Company, Mountain View, CA). Nominal sampling times were used to estimate all pharmacokinetic parameters unless the deviation (scheduled *vs* actual) was 10% (or greater) where the actual time of sample collection was used. The PK parameters reported consist of C_max_ (maximum plasma concentration), AUC_inf_ (area under the concentration time curve based on extrapolation to infinity), AUC_inf_/Dose (dose normalized area under the curve), Cl (clearance, corresponding to the overall rate of elimination of FG-3019 from plasma), V_z_ (volume of distribution during the terminal phase), V_ss_ (volume of distribution during the steady-state), and t_1/2_ (terminal half-life corresponding to the log-linear slope of the observed terminal phase of the concentration-time profile). All PK parameters values were reported to three significant figures (Table S1).

### Compartmental Pharmacokinetic Modeling

A pharmacokinetic (PK) model was designed to describe the time courses of plasma concentrations of FG-3019 (Ab) and its targets CTGF (W) and the N-fragment of CTGF (N). The model assumes that both W and N are constitutively produced at zero-order rates k_W_ and k_N_, and cleared by first-order processes CL_W_ and CL_N_, respectively. They also distribute to peripheral tissues W_T_ and N_T_ at distributional clearance rates CL_dW_ and CL_dN_, respectively. CTGF is cleared from the tissues at the first-order clearance rate CL_WT_. The antibody is cleared from the circulation at the first-order clearance rate CL_Ab_ and distributes to the peripheral tissue compartment Ab_T_ at clearance rate CL_dAb_. Ab binds to the target species W and N at second-order rate constants k_onW_ and k_onN_ and forms complexes AbW and AbN, respectively. The complexes dissociate to single molecule species Ab and W, and Ab and N at first-order rates k_offW_ and k_offN_, respectively. The complexes AbW and AbN distribute to the tissue compartments AbW_T_ and AbN_T_ at the first-order clearance rates CL_dAbW_ and CL_dAbN_, respectively. The complex AbW_T_ is eliminated from the tissues at the first-order clearance rate CL_AbWT_. The binding and dissociation of the antibody, CTGF, and CTGF-N in the tissues were neglected. See the Supplemental section for the mathematical description of the model.

Concentrations were expressed in nM units using the molecular weights of 38 kD for CTGF, 19 kD for N-fragment, and 75 kD for FG-3019. The latter accounts for the two binding sites per molecule of 150 kD IgG protein. The measurements below limit of quantification were ignored. Mean data were used for analysis. All data sets analyzed were fit simultaneously using the maximum likelihood estimator. Precision of the parameters was expressed as percent coefficient of variation. The goodness of fit was assessed by overlaying the observed data with model predictions and from the correlation coefficients between observed and predicted values for each dose group. Nonlinear regression was performed by ADAPT 5 (8).

## Results

### Pharmacokinetics of FG-3019 in Rats

**Fig. 1:**
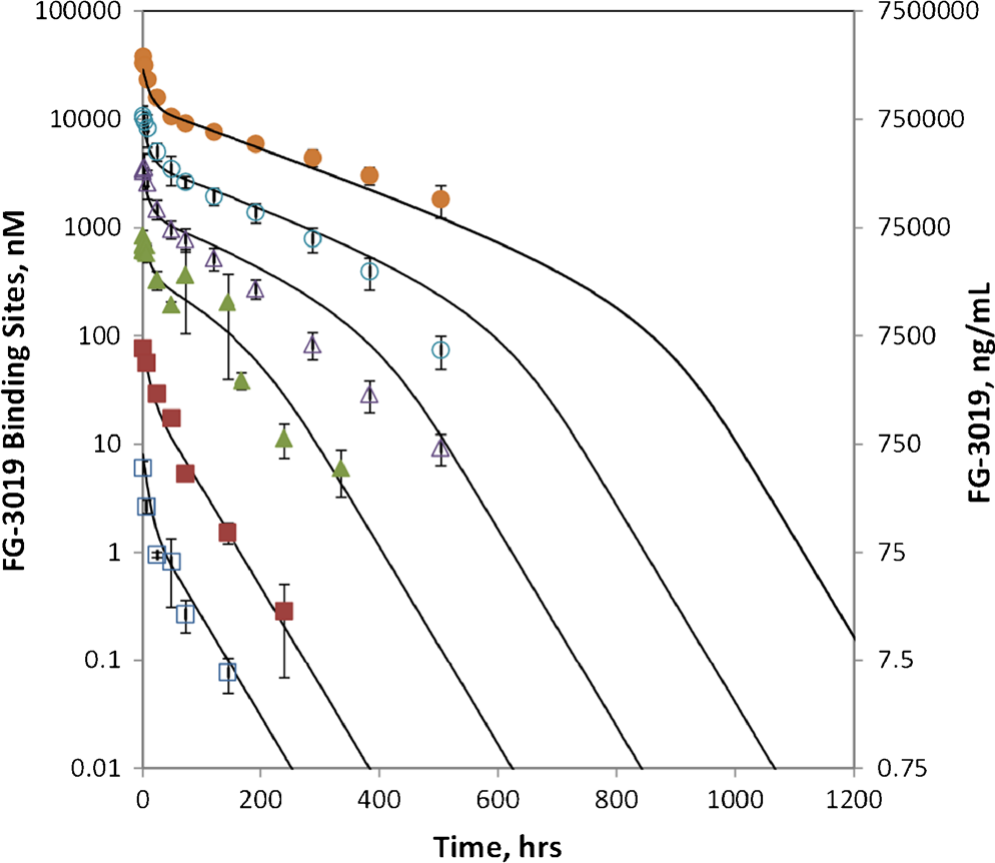
Plasma concentration time courses for FG-3019 in male rats. The mean plasma concentration of FG-3019 is expressed in ng/ml (*right axis*) and in nanomolar binding site concentration (MW = 75,000) (*left axis*). FG-3019 doses administered were 0.03 mg/kg (*n* = 3) (*open squares*), 0.3 mg/kg (*n* = 3) (*solid squares*), 3 mg/kg (*n* = 9) (solid triangles), 10 mg/kg (*n* = 6) (open triangles), 30 mg/kg (*n* = 6) (*open circles*) and 100 mg/kg (*n* = 6) (*solid circles*). Error bars reflect standard deviations. Solid curves are from fits of the data using a pharmacokinetic model.

Preliminary assessment of the PK of FG-3019 in several species indicated relatively rapid clearance and a short terminal half-life (not shown). To fully characterize the PK of FG-3019, its plasma concentration in rats was measured following IV doses ranging from 0.03 to 100 mg/kg (Fig. 1). The apparent terminal phase was more rapid at low than at high dose, which is reflected in the calculated half-life (T_1/2_) values that ranged from 1.36 days at 0.03 mg/kg to 7.31 days at 100 mg/kg (Table S1). Clearance (Cl) decreased with increasing FG-3019 dose from 90.6 ml/kg/day to 8.9 ml/kg/day over the 0.03–100 mg/kg dose range. Consistent with the dose-dependent decrease in Cl, the dose-normalized AUC (AUC_inf_/Dose) increased with dose level. The non-linearity of the rat pharmacokinetic parameters with the dose of FG-3019 indicates a role of a saturable process in the elimination mechanism.

### Effect of FG-3019 on Circulating CTGF in Rats

**Fig. 2:**
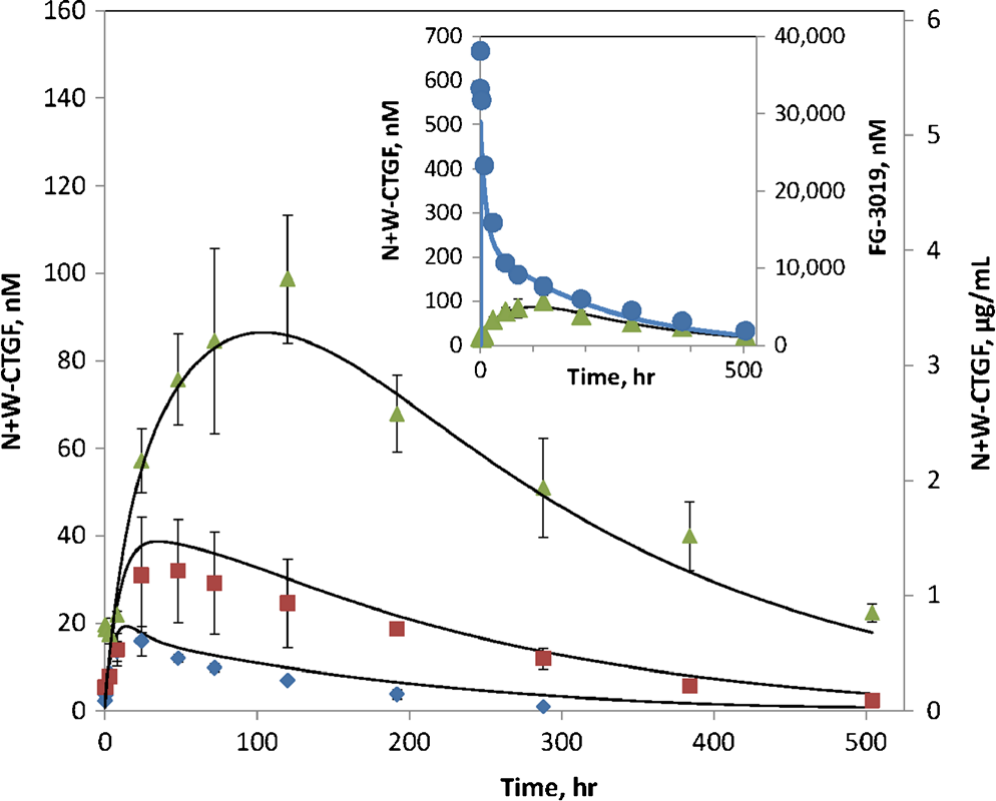
Rat plasma concentrations of endogenous CTGF following administration of FG-3019. Male rats were administered FG-3019 at 10 mg/kg (*diamonds*), 30 mg/kg (*squares*) or 100 mg/kg (*triangles*). CTGF was measured using the N+W-CTGF assay as described in the text. Error bars are standard deviations of the means of three animals. Concentrations of intact CTGF measured with the W-CTGF assay are below 5 ng/ml and are not shown. Solid curves are fits obtained from pharmacokinetic modeling. *Inset*: concentrations of FG-3019 in rats dosed at 100 mg/kg (circles,*right axis*) are shown with the corresponding CTGF concentrations (triangles,*left axis*).

Endogenous CTGF plasma concentrations were essentially unchanged upon dosing with FG-3019. Pre-dose concentrations were uniformly below the 5.6 ng/ml lower limit of quantitation. For the high dose group there was only one time point (0.5 h) for which all three test animals had quantifiable W-CTGF concentrations, averaging 10 ± 3 ng/ml (not shown). In contrast, plasma concentrations of CTGF-N detected by the N+W-CTGF assay rose substantially after dosing with FG-3019 (Fig. 2). Both the maximum level and the time to maximum N+W-CTGF level were dose dependent, with C_max_ N+W-CTGF concentrations of 32, 77 and 197 ng/ml, and T_max_ times of 24, 48 and 120 h for the 10, 30 and 100 mg/kg FG-3019 doses, respectively. CTGF concentrations for the 0.03, 0.3 and 3 mg/kg dose level were not determined. Since CTGF-whole concentrations were unaffected by FG-3019 dosing, the increase in signal measured by the N+W-CTGF assay corresponds to accumulation in the plasma of CTGF-N. It should be noted that the CTGF-N in circulation after dosing with FG-3019 is expected to be mostly complexed with FG-3019 due to the excess concentration of circulating antibody.

### Pharmacokinetics of CTGF

**Fig. 3:**
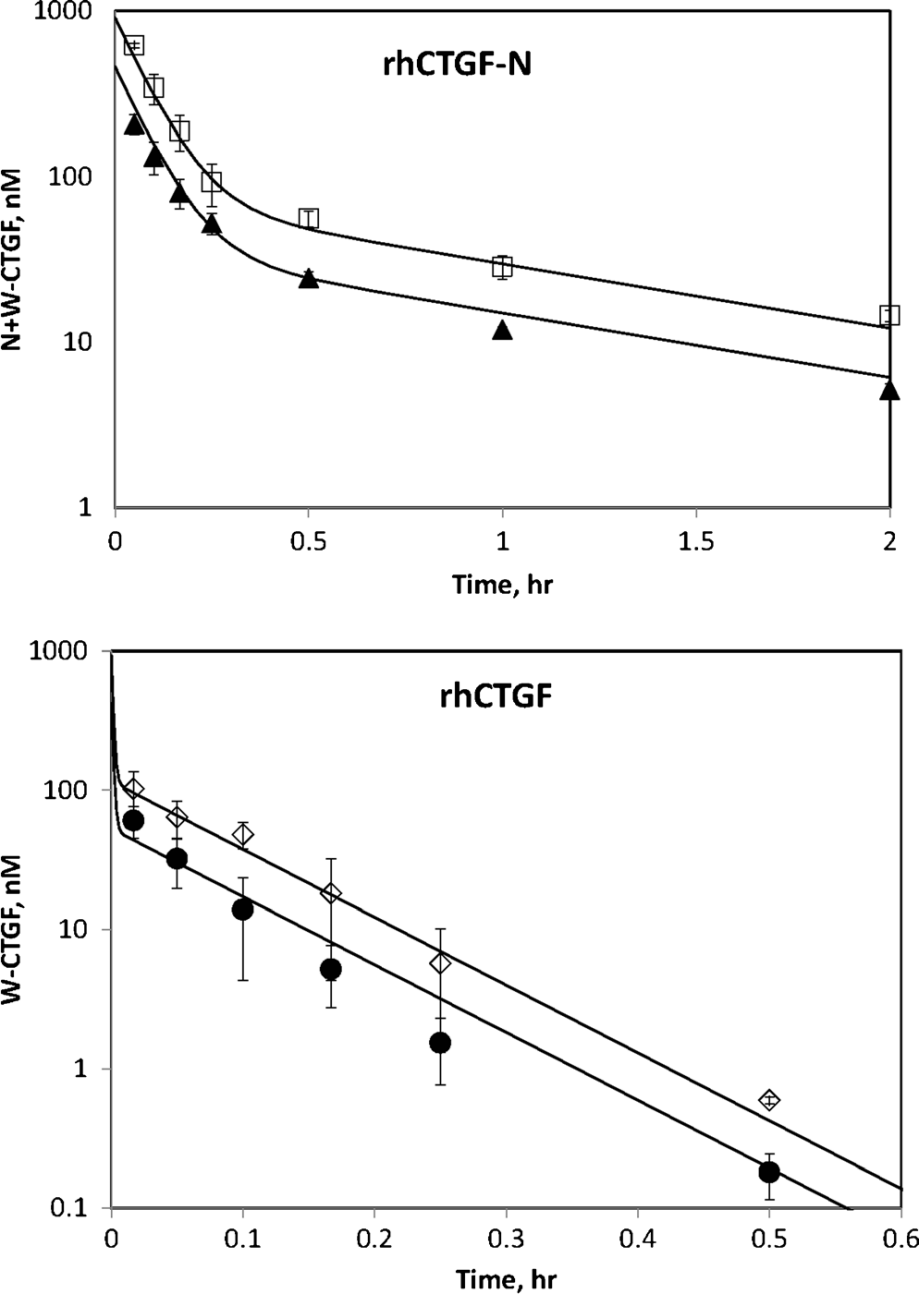
Plasma Concentration Time Courses of Recombinant Human CTGF in Male Rats. Top panel: rhCTGF-N was administered at 0.38 mg/kg (solid triangles) and 0.8 mg/kg (*open squares*). CTGF was measured using the N+W-CTGF assay. Bottom panel: rhCTGF was administered at 0.76 mg/kg (*solid circles*) and 1.6 mg/kg (*open diamonds*). CTGF was measured using the W-CTGF assay. Error bars are standard deviations from 3 animals per group. Solid curves were obtained by a pharmacokinetic model.

The pharmacodynamic effect of FG-3019 on circulating CTGF-N concentration raised questions about the source of plasma CTGF-N, the mechanism for the post FG-3019 dose increase, and the absence of this effect for CTGF-W. To begin to address these questions and develop a PK/PD model for FG-3019 we decided to evaluate the kinetics of CTGF itself. We found that disappearance of recombinant human CTGF-N from the blood following an intravenous dose (Fig. 3) was rapid and biphasic, with average terminal phase half-life of 43.5 min. The initial phase of disappearance of plasma CTGF-whole following intravenous injection was too fast to allow for non-compartmental assessment. The terminal phase had a half-life of 3.3 min, 13-fold faster than the terminal phase half-life of CTGF-N. Thus, the elimination kinetics of both forms of CTGF were clearly much faster than the kinetics observed for FG-3019.

### Effect of Recombinant Human CTGF on FG-3019 Pharmacokinetics in Rats

**Fig. 4:**
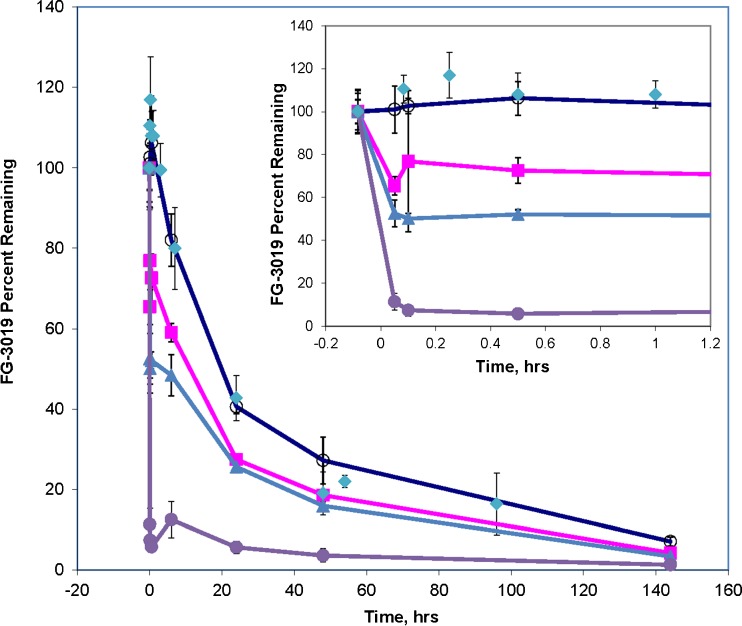
Effect of Co-administered CTGF on FG-3019 pharmacokinetics. The amount of FG-3019 (expressed as percent of initial level remaining) is shown *versus* time after administration of CTGF or other components. Components co-administered were: buffer (*open circles*); CTGF-N (*solid diamonds*); CTGF at 0.5:1 molar ratio with FG-3019 (*solid squares*); CTGF at 1:1 molar ratio with FG-3019 (*solid triangles*); CTGF at 2:1 molar ratio with FG-3019 (*solid circles*). FG-3019 was administered at −10 min. CTGF was added at time zero.Error bars reflect the standard deviation of mean values.

In rats, rhCTGF-N is eliminated from circulation rapidly, but the endogenous N-fragment of CTGF accumulates after dosing with FG-3019. This suggests that accumulation may be due to much slower elimination kinetics for the complex of CTGF-N with FG-3019 than for free CTGF-N. Similar accumulation of other soluble target/antibody complexes has been reported (9,10). In contrast, the intact form of endogenous CTGF did *not* accumulate in the circulation after dosing with FG-3019, despite the affinity of the antibody for CTGF (K_d_ = 0.1–0.2 nM, unpublished radioimmunoassay results). This indicates either that the rate of production of CTGF-whole into the circulation is too low to result in significant accumulation of CTGF/FG-3019 complexes, or the complex formed between CTGF and FG-3019 is eliminated too rapidly to allow for significant accumulation. To evaluate these two possibilities we chose to estimate the rate of elimination of FG-3019/CTGF complexes by determining the effect of co-administered CTGF on FG-3019 pharmacokinetics. Administration of CTGF-N to rats previously dosed with FG-3019 had no significant effect on the level of FG-3019, or the rate of disappearance of FG-3019 from the circulation (Fig. 4). However, co-administration of intact CTGF had a profound impact on FG-3019 kinetics.

As shown in Fig. 4, dosing with CTGF caused an extremely rapid decrease of plasma FG-3019 concentration. This effect was CTGF dose-dependent, such that 23%, 50% and 93% decreases in FG-3019 concentration 6 min after injecting CTGF were seen at the low, middle and high CTGF:FG-3019 ratios, respectively. After the initial rapid drop in FG-3019 concentration the elimination rate of FG-3019 was indistinguishable from the rate of FG-3019 elimination in animals that were not injected with CTGF. It appeared that the injected rhCTGF was complexing with FG-3019 and this complex was then eliminated from the blood in the same rapid fashion observed for rhCTGF in the absence of FG-3019. Interestingly, the impressive decrease in FG-3019 concentration triggered by administration of CTGF in 2:1 stoichiometry to FG-3019 was followed six hours later by a small, but reproducible increase in FG-3019 concentration. This may have resulted from dissociation of the antibody from CTGF, or from antibody recycling.

**Fig. 5:**
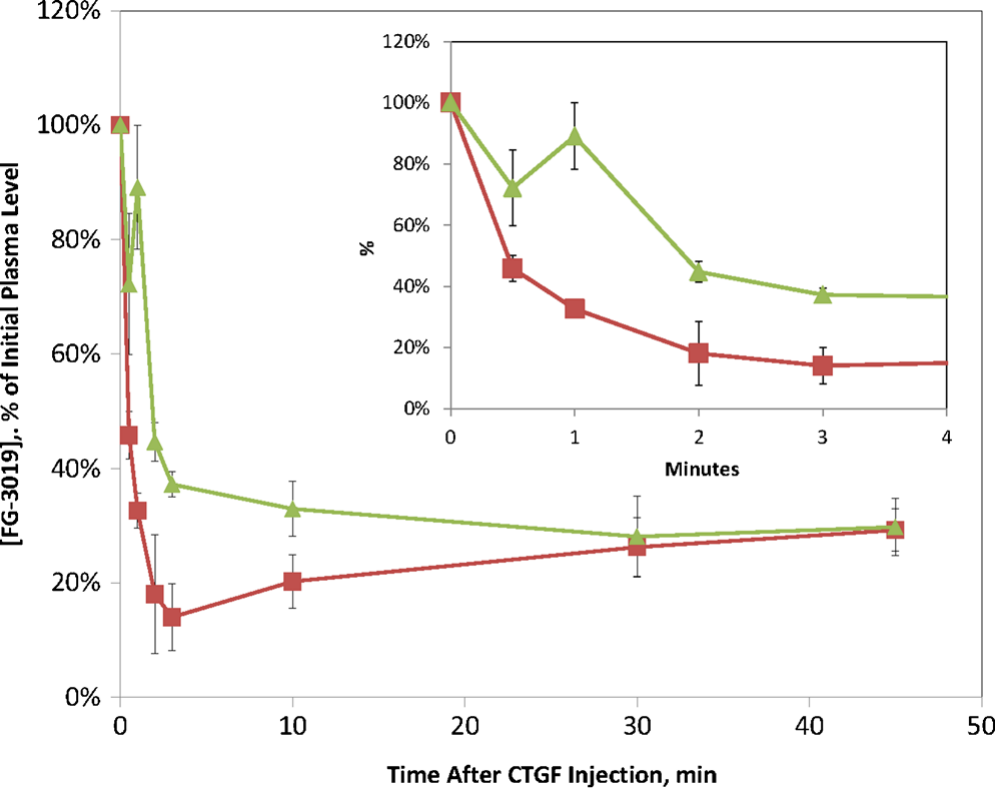
Effect of Co-administered RAP on CTGF mediated elimination of FG-3019. The level of FG-3019 (expressed as percent of initial level) is shown *versus* time after administration of CTGF (*squares*), or RAP (9 mg) immediately followed by CTGF (*triangles*). CTGF was administered at a 2:1 molar ratio to FG-3019. CTGF or (RAP + CTGF) was administered at time zero, 10 min after FG-3019. Error bars reflect standard deviation of mean values.

CTGF has been reported to bind to the receptor LRP1, which we thought might be responsible for the rapid elimination of CTGF and CTGF/FG-3019 complexes from the blood. We therefore tested the effect of an inhibitor of LRP1 binding activity, the receptor associated protein (RAP), on CTGF’s clearing activity toward FG-3019 (7,11). Co-administration of 9 mg RAP per animal significantly reduced the impact of CTGF on FG-3019 elimination kinetics, consistent with involvement of LRP-1 (and/or LRP2/megalin) in the CTGF mediated antibody clearance (Fig. 5).

The kinetic data for FG-3019, rhCTGF and rhCTGF-N together with the effect of CTGF on FG-3019 PK and the pharmacodynamic effect of FG-3019 on CTGF concentrations indicate that when CTGF and CTGF-N are secreted from tissues they share different fates upon encountering FG-3019. CTGF-N binds to FG-3019 and takes on the relatively slow elimination kinetics of free antibody while intact CTGF binds to FG-3019 and causes the antibody to be rapidly eliminated, presumably through the same pathway as free CTGF. To explore this model further we defined a kinetic model and fit the relevant kinetic data as described below.

### Pharmacokinetic Modeling of Target-Mediated Elimination of FG-3019

**Fig. 6:**
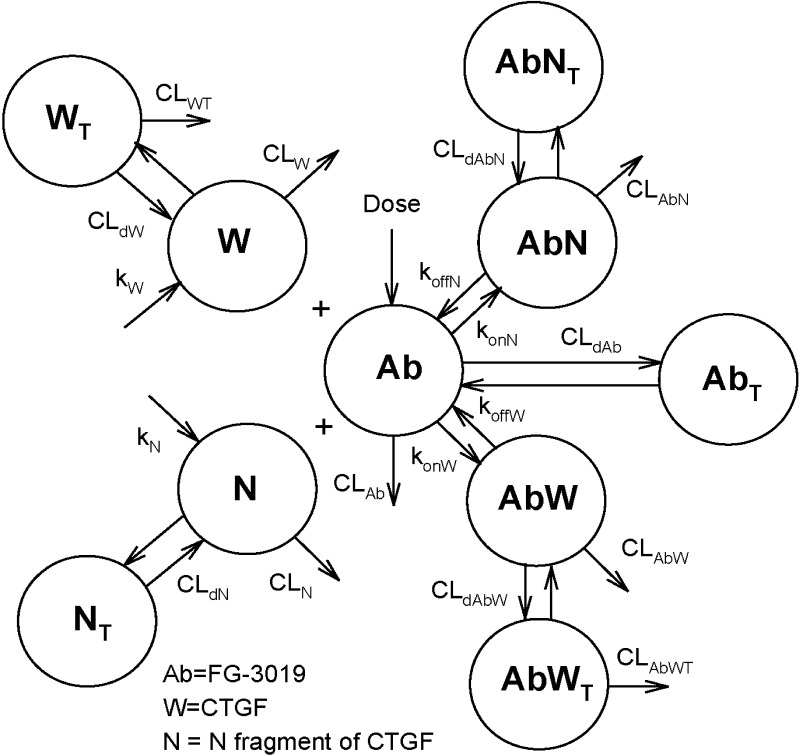
Schematic diagram of the PK model of target mediated disposition of FG-3019. The model assumes that both CTGF (W) and CTGF-N (N) are constitutively produced at zero-order rates k_W_ and k_N_, and cleared by first-order processes CL_W_ and CL_N_, respectively. They also distribute to peripheral tissues W_T_ and N_T_ at distributional clearance rates CL_dW_ and CL_dN_, respectively. CTGF is cleared from the tissues at the first-order clearance rate CL_WT_. The antibody, FG-3019, is cleared from the circulation at the first-order clearance rate CL_Ab_ and distributes to the peripheral tissue compartment Ab_T_ at clearance rate CL_dAb_. Ab binds to the target species W and N at second-order rate constants k_onW_ and k_onN_ and forms complexes Ab_W_ and Ab_N_, respectively. The complexes dissociate to single molecule species Ab and W, and Ab and N at first-order rates k_offW_ and k_offN_, respectively. The complexes Ab_W_ and Ab_N_ distribute to the tissue compartments Ab_WT_ and Ab_NT_ at the first-order clearance rates CL_dAbW_ and CL_dAbN_, respectively. The complex Ab_WT_ is eliminated from the tissues at the first-order clearance rate CL_AbWT_. The binding and dissociation of the antibody, CTGF, and CTGF-N in the tissues were neglected.

A pharmacokinetic model (Fig. 6) was designed to account for the data presented above. For simplicity, we elected to confine antibody/CTGF binding reactions in this model to the central compartment (*i.e*. plasma), with uptake and elimination of the complex between antibody and CTGF through a tissue compartment. This is in distinction to a model where antibody distributes into tissues, binds to locally produced CTGF, and then undergoes target-mediated elimination as an antibody/CTGF complex without returning to the central compartment. In this model, the following steps constitute the target-mediated clearance pathway: binding of antibody (Ab) to CTGF (W) in the central compartment, distribution of the Ab-CTGF complex to tissues (AbW_T_) at rate CL_dAbW_, followed by elimination of the Ab-CTGF complex from the tissue compartment at a rate CL_AbWT_. The time course for the initial rapid phase of rhCTGF disappearance (Fig. 3) could not be fit due to lack of data points. Therefore, the *t* = 0 plasma concentration of CTGF was set equal to the extrapolated *t* = 0 concentration from the N-fragment kinetics analysis, and the initial rapid phase for CTGF was forced to be very rapid in the modeling by setting CL_W_ = 10 L/h.

The PK model involved a large number of parameters that could not be resolved from the available data. To avoid problems with the model identifiability, some parameters were assumed to be equal and some processes were considered as very rapid. The distributional clearances for W and the complex AbW were assumed to be fast and set as CL_dW_ = CL_dAbW_ = 10 L/h with V_AbWT_ = V_WT_. This assumption was based upon the observation that exogenously added CTGF resulted in very rapid elimination of FG-3019 from circulation (Fig. 4) and the very rapid clearance of rhCTGF (Fig. 3). The binding of FG-3019 to target was considered rapid. To avoid introducing a rapid binding approximation that would require overly complex equations (12) we enforced this assumption by setting the dissociation rate constants k_offW_ = k_offN_ = 100 h^−1^. We assumed that the complex of FG-3019 bound to N-fragment follows the distribution kinetics of free FG-3019, CL_dAbN_ = CL_dAb_ and V_AbNT_ = V_AbT_. This assumption was based upon the observation that exogenously added CTGF-N did not result in very rapid elimination of FG-3019 from circulation (Fig. 4). The tissue clearance rates of W and AbW were set equal, CL_WT_ = CL_AbWT_. Also the clearance rates of W and N from the plasma were assumed to be the same, CL_W_ = CL_N_. While this assumption is not strictly correct based on the data shown in Fig. 3, the plasma clearance of W and N are so much faster than free Ab that the difference between CL_W_ and CL_N_ should not matter. The non-target mediated clearance rates from the plasma compartment of antibody-bound forms of CTGF were set equal to the free antibody clearance rate, CL_Ab_ = CL_AbW_ = CL_AbN_. It was also assumed that endogenous and recombinant forms of CTGF are kinetically identical.

Three datasets were simultaneously analyzed. The first data set comprised the observed CTGF plasma concentrations following injections of rhCTGF or CTGF-N (Fig. 3). The assays do not detect the endogenous rat CTGF; therefore, both W_0_ and N_0_ were set to 0. The second data set comprised the plasma concentrations of FG-3019 (total of free and CTGF-complexed forms) following administration of the antibody (Fig. 1). Plasma samples for measurements were collected for up to 504 h. The third data set was comprised of the total combined plasma concentrations of endogenous CTGF-N plus CTGF (free and antibody-bound) following injection of FG-3019 (Fig. 2). We did not include data for the effect of exogenous rhCTGF on FG-3019 kinetics (Fig. 4) because the model treats FG-3019 as a monovalent species with 75 kDa molecular weight, whereas rhCTGF added to FG-3019 at stoichiometric ratios will form significant amounts of complex having a 2-to-1 ratio of rhCTGF to the bivalent 150 kDa FG-3019 molecule. We also did not include data for the inhibition by RAP of the CTGF clearing effect (Fig. 5) because we lacked data on RAP kinetics in rat.

Model parameters obtained from data fitting are listed in Table I and graphical results are shown as solid curves superimposed onto the experimental data in Figs. 1, 2 and 3. The model fits of the antibody plasma concentration (combined free antibody and antibody/CTGF complexes) shown in Fig. 1 exhibit patterns characteristic of target-mediated disposition (13). The initial phase corresponds to rapid tissue distribution. For high doses, this is followed by a plateau due to saturation of the target-mediated clearance pathway, which is followed by a faster terminal elimination phase as the target-mediated pathway becomes predominant at lower antibody concentration (14). The fits of combined plasma concentrations for species detected by the N+W-CTGF assay (Fig. 2) are dominated by the total of CTGF-N and antibody/CTGF-N complex. Calculated CTGF is included in the sum since the immunoassay detects this species, but concentrations of CTGF are in fact negligible. The combined concentration of free and bound CTGF-N starts at a low baseline value, reaches a peak and returns to the baseline. Such behavior is characteristic for target-mediated drug disposition when the clearance of the drug-target complex is slower than the clearance of the free target (15). Only the lowest dose data at late time points were slightly over-predicted with r^2^ = 0.94, with the remaining data well described by the model (r^2^ = 0.97–0.98).

**Table I:** Calculated Model Parameter Values and Their Coefficients of Variation

Parameter	Estimate	CV%
V, L	0.01512	6.3
CL_W_, L/h	0.09243	3.7
V_WT_,L	0.1160	13.0
CL_WT_, L/h	1.380	9.5
CL_dW_, L/h	10	FIXED
CL_N_,L/h	0.09243^a^	NA
V_NT_, L	0.04458	11.5
CL_dN_, L/h	0.07998	9.4
K_DW_, nM	23.97	18.0
K_DN_, nM	51.21	15.0
k_offW_, h^−1^	100	FIXED
k_offN_, h^−1^	100	FIXED
C_W0_, nM	0.02591	15.6
C_N0_, nM	0.6572	10.6
CL_Ab_, L/h	0.0001321	6.4
V_AbT_, L	0.01363	12.5
CL_dAb_, L/h	0.0005692	27.7
CL_AbW_, L/h	0.0001331^b^	NA
CL_dAbW_, L/h	10	FIXED
CL_AbN_, L/h	0.0001321^b^	NA
CL_dAbN_, L/h	0.0005692^c^	NA
CL_AbWT_, L/h	1.380^d^	NA
k_W_, nmol/h	0.03382^e^	13.5
k_N_, nmol/h	0.06075^e^	10.2

^a^CL_N_ = CL_W_

^b^CL_Ab_ = CL_AbW_ = CL_AbN_

^c^CL_dAbN_ = CL_dAb_

^d^CL_AbWT_ = CL_WT_

^e^secondary parameter

The estimated central volume V = 50.4 ml/kg (for a 0.3 kg animal) is close to the reported rat plasma volume of 39.6 ml/kg (16). The estimated central compartment clearance rate for FG-3019, CL_Ab_, is 0.1355 ml/h (0.45 ml/h/kg for a 0.3 kg animal) compared with a published plasma clearance rate of 0.8 ml/h/kg for ^125^I-labeled human IgG in rats (17). Estimated baseline plasma concentrations of CTGF and CTGF-N were 0.0259 and 0.657 nM (0.98 and 12.5 ng/ml), respectively, whereas the measured concentrations at baseline were below the LLOQ of 5.6 ng/ml for CTGF and below the LLOQ of 11 ng/ml for the sum of CTGF and CTGF-N. Calculated production rates of CTGF and CTGF-N (k_W_ and k_N_) were 0.034 and 0.061 nmol/h, respectively. Since CTGF-N is derived from CTGF, the total estimated rate of CTGF production per animal is the sum of these rates, 0.095 nmol/h, which is equivalent to 0.29 mg/kg/day. The model-estimated antibody binding constants (K_d_) for CTGF and CTGF-N were 24 and 51 nM, respectively. The value for CTGF is 120-fold larger than the K_d_ obtained from *in vitro* studies employing ^125^I-labeled CTGF (unpublished data). The value for CTGF-N is 3-fold larger than measured by a surface plasmon resonance kinetic method. However, similar discrepancies have been reported for other antibodies, suggesting that *in vitro* K_d_ measurements in buffer using radioiodinated human CTGF overestimate the effective *in vivo* binding affinity of endogenous rat CTGF (18).

### Dose-Dependence of Target-Mediated Elimination

**Fig. 7:**
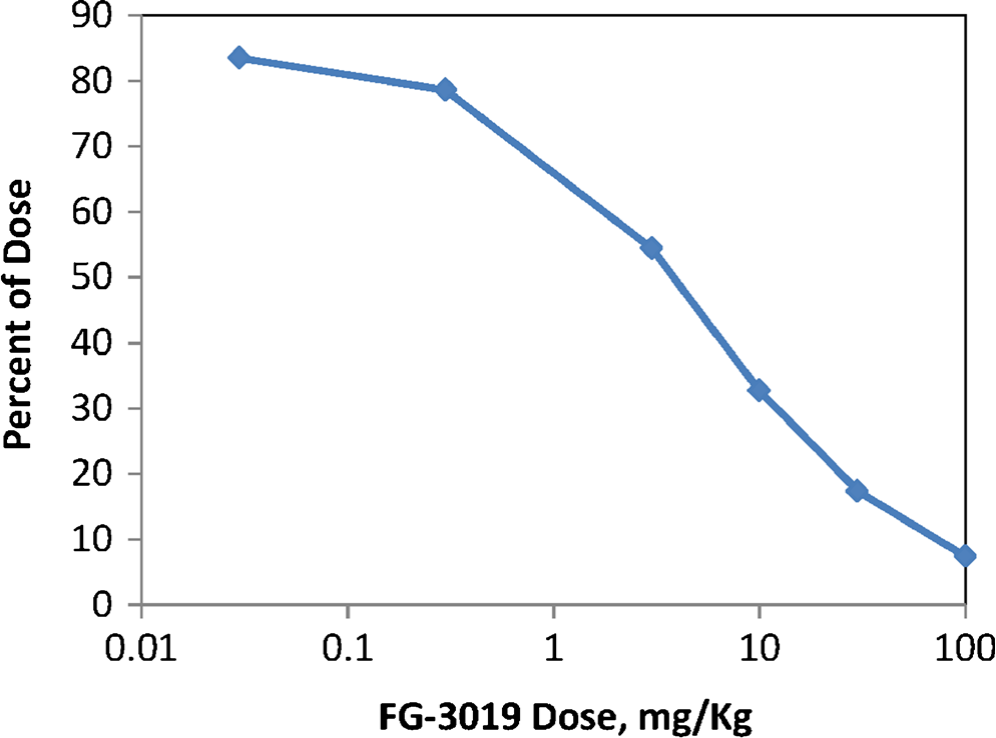
Dose-dependence of target-mediated elimination of FG-3019. The percent target-mediated elimination was estimated by modeling rates of elimination of FG-3019 from tissue and central compartments.

The degree to which target-mediated antibody elimination is responsible for the overall clearance of FG-3019 was estimated by simulating the elimination rate of FG-3019/CTGF complex from the tissue compartment over time and comparing this to the simulated combined central compartment elimination rates for all FG-3019 containing species. The integrated areas under the simulated curves correspond to the amount of antibody eliminated. Figure 7 shows the percentage of antibody that is cleared by the target-mediated pathway. For doses at or below 3 mg/kg, target-mediated clearance is the major pathway for antibody elimination. In 300 g rats, an IV dose of 3 mg/kg FG-3019 corresponds to a C_max_ plasma concentration of approximately 1 μM. Thus, at plasma concentrations below 1 μM, target-mediated elimination dominates FG-3019 clearance, while at higher concentrations it contributes less. At the highest dose tested (100 mg/kg), 7.4% of the FG-3019 dose was eliminated by the target-mediated pathway. In a subject with disease that may be producing and shedding more CTGF, target-mediated elimination would be expected to play a larger role in the clearance of FG-3019. Similarly, the terminal elimination rate would be expected to be higher, in proportion to the increased rate of CTGF shedding.

### Rat Tissue Distribution of ^125^I-FG-3019 and Effect of CTGF

The complex between FG-3019 and CTGF is rapidly eliminated from the blood. To identify the site(s) where rhCTGF causes FG-3019 to be redistributed, radio-iodinated FG-3019 ([^125^I]-FG-3019) was prepared and examined for its distribution in rat tissues in the absence and presence of rhCTGF using quantitative whole body autoradiography (QWBA).

Gamma counting of urine, feces and cage wipe materials from animals dosed with [^125^I]-FG-3019 alone showed that 0.8%, 1.7% and 23.7% of the dosed radioactivity was excreted from animals euthanized at 2, 6 and 24-h post dose, respectively. Concentrations of [^125^I]-FG-3019 determined by gamma counting in blood and plasma are shown in Table II. [^125^I]-FG-3019 concentrations were approximately half as high in the blood as plasma, indicating that [^125^I]-FG-3019 is restricted to the non-cellular portion of blood. The rat dosed with CTGF had 6.2- and 3.9-fold lower [^125^I]-FG-3019 concentrations in plasma and blood, respectively, than the corresponding rat which was not dosed with CTGF. This large decrease in [^125^I]-FG-3019 concentration is consistent with the effect of CTGF on plasma concentrations of non-radioactive FG-3019 observed in the studies described previously. The blood/plasma ratio (0.708) in the rat dosed with CTGF was higher than in the animals not dosed with CTGF, suggesting that the CTGF/FG-3019 complex associates slightly more with the cellular components of blood than FG-3019 in the absence of CTGF.

**Table II:** Tissue Concentrations of Radioactivity After [^125^I]-FG-3019 Administration

Tissue type	Tissue	Mean - μg equivalents/g tissue					
		Rat # 1	Rat # 2^a^	Rat # 3	Rat # 4	Rat # 5	Ratio
		0.25 h	0.25 h	2 h	6 h	24 h	Rat 2/Rat 1
Vascular/ lymphatic	Blood (by QWBA)	113.2	24.0	105.9	124.7	53.4	**0.21**
	Bone marrow	35.6	34.5	25.1	38.9	19.5	0.97
	Lymph node	6.4	4.4	19.0	14.4	9.8	0.70
	Spleen	27.0	30.9	24.5	26.3	15.5	**1.15**
	Thymus	10.4	2.8	9.7	11.9	7.7	**0.27**
Excretory/ metabolic	Bile (in duct)	NI	52.3	115.5	NI	21.3	NC
	Kidney cortex	29.8	56.1	23.8	27.8	14.0	**1.88**
	Kidney medulla	35.1	24.9	28.7	31.6	15.9	0.71
	Liver	33.9	198.9	19.9	20.6	11.5	**5.87**
	Urinary bladder	13.0	3.3	26.1	9.9	<2.0	**0.25**
	Urinary bladder (contents)	6.3	<2.0	24.1	63.8	11.1	**<0.32**
Central nervous system	Brain (cerebrum)	2.9	<2.0	3.2	3.7	<2.0	<0.68
	Brain (cerebellum)	2.8	<2.0	3.7	5.4	<2.0	<0.70
	Brain (medulla)	<2.0	<2.0	2.1	2.0	<2.0	NC
	Spinal cord	4.7	<2.0	3.0	3.4	<2.0	<0.42
Endocrine	Adrenal cortex	25.8	257.7	35.9	34.9	25.2	**10.00**
	Adrenal medulla	44.3	136.0	53.4	51.7	29.2	**3.07**
	Pituitary gland	24.3	14.1	26.5	20.6	12.1	0.58
	Thyroid	15.1	7.4	14.8	25.9	13.5	0.49
Secretory	Harderian gland	3.5	<2.0	6.4	9.0	9.4	<0.57
	Pancreas	8.6	5.1	6.3	9.7	7.7	0.59
	Salivary gland	5.2	2.7	9.5	11.4	9.9	0.52
Fatty	Adipose (brown)	25.6	10.4	32.4	40.6	19.4	0.41
	Adipose (white)	6.2	2.5	<2.0	4.7	2.4	0.40
Dermal	Skin (non-pigmented)	3.4	2.7	5.2	7.4	7.4	0.79
Reproductive	Epididymis	2.9	<2.0	6.0	10.5	11.3	<0.69
	Prostate gland	4.9	<2.0	8.0	7.4	3.9	<0.40
	Seminal vesicles	2.5	<2.0	3.8	7.3	<2.0	<0.81
	Testis	3.0	2.2	12.1	17.6	13.1	0.75
Skeletal/ muscular	Bone	3.9	5.1	5.6	3.8	2.5	**1.33**
	Heart	30.3	9.8	35.5	29.4	20.1	**0.32**
	Skeletal muscle	2.1	<2.0	2.8	2.4	<2.0	<0.95
Respiratory	Lung	75.2	22.9	65.0	78.2	33.8	**0.31**
Alimentary canal	Cecum	2.7	<2.0	6.3	24.6	8.1	<0.74
	Cecum (contents)	<2.0	<2.0	<2.0	2.6	<2.0	NC
	Large intestine	4.6	2.4	4.6	13.1	9.9	0.52
	Large intestine (contents)	<2.0	<2.0	<2.0	3.2	<2.0	NC
	Stomach (gastric mucosa)	8.8	6.8	12.1	30.2	5.5	0.77
	Stomach (contents)	<2.0	3.4	8.4	31.7	<2.0	NC
	Small intestine	5.1	<2.0	17.2	34.4	7.9	<0.39
	Small intestine (contents)	<2.0	<2.0	8.1	16.3	<2.0	NC
Ocular	Eye (uvea)	3.8	<2.0	10.1	11.5	9.5	<0.52
	Eye (lens)	<2.0	<2.0	<2.0	<2.0	<2.0	NC

^a^This animal received a 5 mg/kg dose of CTGF at approximately 10 min after [^125^I]-FG-3019 administration

NI = Tissue not collected during sectioning

NC = Not calculable

Ratios of Rat 2/Rat 1 concentrations are bolded for values greater than 1.0 and less than 0.33.

**Fig. 8:**
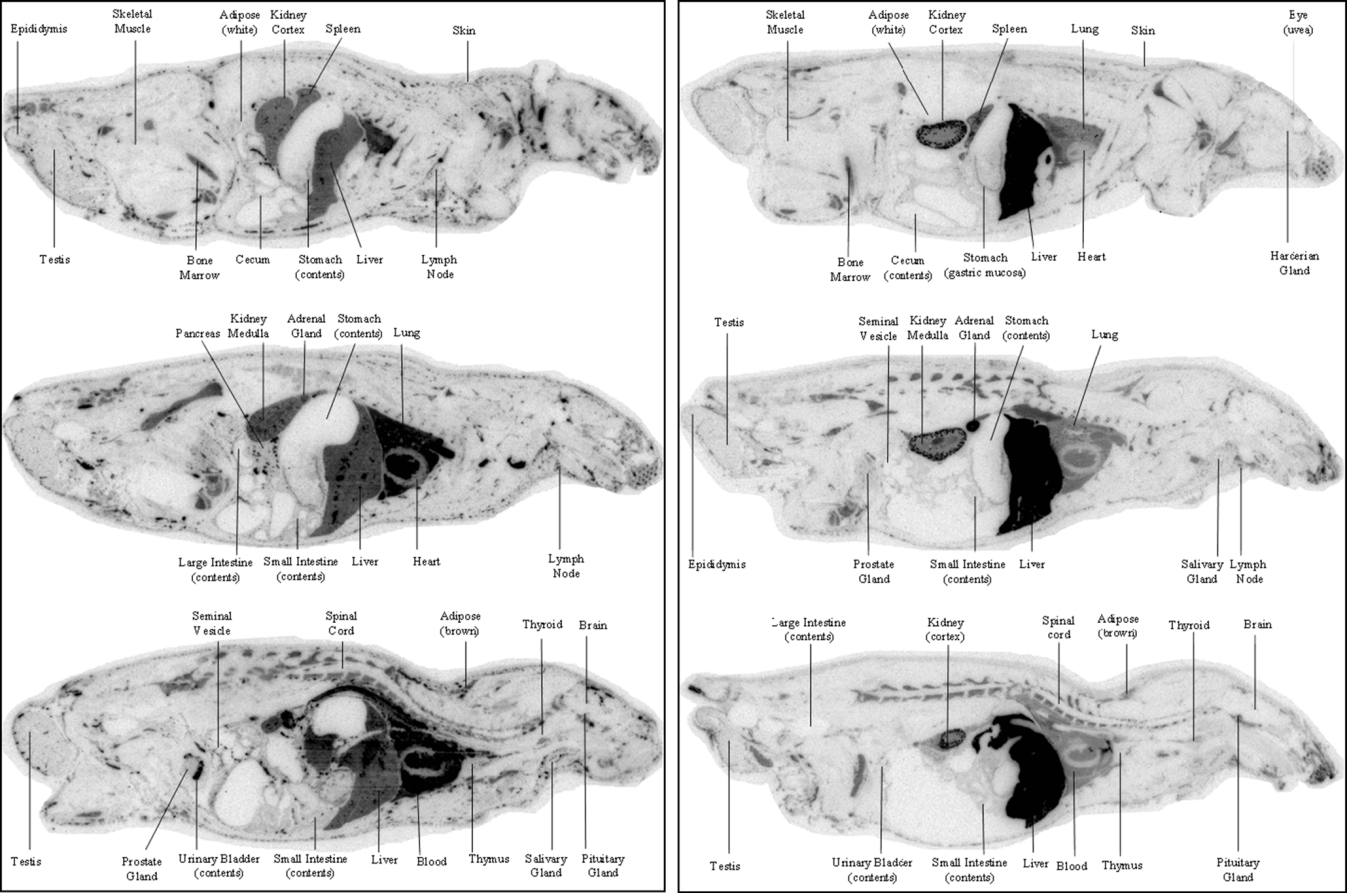
Distribution of [^125^I]-FG-3019 at 15 min after administration without or with addition of CTGF. *Left panel*: without administration of CTGF. *Right panel*: with administration of CTGF 5 min before euthanasia.

Whole-body autoradiograms showing patterns of radioactivity distribution in tissues from rats euthanized at 0.25 h are illustrated in Fig. 8. Tissue concentrations of radioactivity are listed in Table II. Note that animals were not perfused prior to QWBA, so tissue concentrations of radioactivity include radioactivity in the blood content of each tissue. Radioactivity was widely distributed in rats dosed with [^125^I]-FG-3019 alone (rat #1, 3, 4 and 5). The highest concentrations of radioactivity (>50 μg equivalent/g) were found in blood, lung and adrenal medulla. Concentrations in the central nervous system, bone, skeletal muscle, white fat, and eye lens were the lowest of all tissues (≤6.2 μg equiv/g). Most tissues had relatively high concentrations of drug-derived radioactivity at 24 h post dose and concentrations ranged from 53.4 μg equivalent/g in blood to 2.4 μg equivalent/g in white fat. The tissues of the central nervous system, seminal vesicles, skeletal muscle, eye lens, urinary bladder, and the contents of the alimentary canal had concentrations of radioactivity below the lower limit of quantitation at 24 h post-dose.

Tissue concentrations of radioactivity in the rat given CTGF 10 min after the dose of [^125^I]-FG-3019 were very different from those dosed with [^125^I] -FG-3019 alone. Most notable were the large fold increases in liver (5.87-fold), adrenal cortex (10-fold), adrenal medulla (3.07-fold), and kidney cortex (1.88-fold). The tissue-to-blood radioactivity concentration ratios for liver, adrenal cortex, adrenal medulla and kidney cortex in the rat co-administered CTGF were 8.3, 10.7, 5.7 and 2.23, respectively, indicating extensive uptake of the [^125^I] -FG-3019/CTGF complex by these organs. Bone marrow and spleen also had high tissue-to-blood concentration ratios of 1.44 and 1.29, respectively, in the rat treated with CTGF. In summary, co-administration of CTGF causes FG-3019 to distribute out of the blood and predominantly into liver, adrenal and kidney.

### Immunohistochemical Analysis

**Fig. 9:**
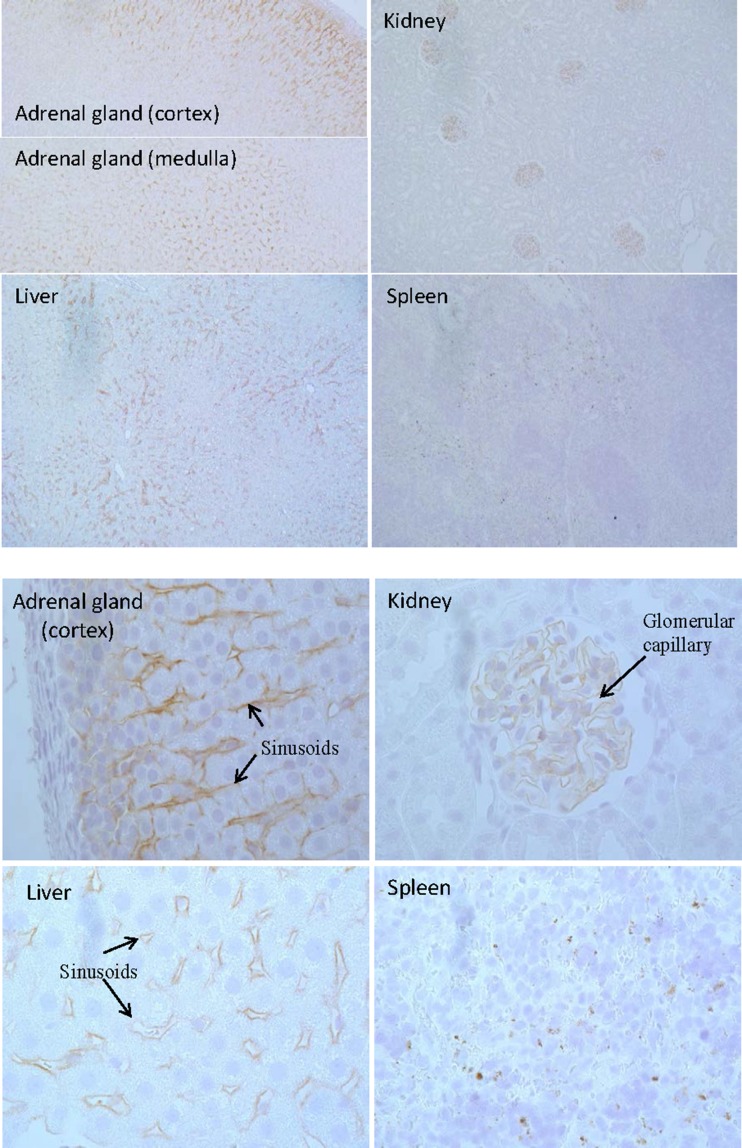
Immunohistochemical Localization of FG-3019. Tissue sections stained for FG-3019 are shown at 10× (upper panel) and 60× (lower panel) magnification for adrenal gland (cortex and medulla), liver, kidney (cortex) and spleen (red pulp) 5 min after IV administration of CTGF to rats dosed 10 min previously with FG-3019. Tissue sections from animals dosed with vehicle after FG-3019 instead of CTGF exhibited no staining for FG-3019.

To explore which cells might take up CTGF/antibody complexes, immunohistochemical analysis was conducted on several tissues (liver, kidney, adrenal, spleen, lung and heart) from rats administered FG-3019 alone or with CTGF. In animals treated with FG-3019 alone no staining for FG-3019 was seen in any of the organs 15 min after injection, which is consistent with the slow penetration of IgG into tissues and the removal of blood by perfusion prior to tissue fixation (not shown). In animals treated with FG-3019 in combination with CTGF, staining for FG-3019 was observed in the sinusoids of adrenal glands and liver and lining the capillaries of kidney glomeruli (Fig. 9). Sparse staining was also observed in the red pulp of the spleen. In contrast with the results in liver, adrenal glands, kidney and spleen, co-administration of rhCTGF did not modify FG-3019 distribution to the heart and lung: no staining for FG-3019 was seen in the heart and lung of animals treated with FG-3019 only, or with a combination of FG-3019 and CTGF (not shown).

## Discussion

In rats, as well as monkeys and humans (data not shown), FG-3019 exhibited non-linear, saturable kinetics where higher doses of FG-3019 resulted in longer apparent terminal half-lives, slower clearance and disproportionately higher plasma exposure (19). Apparent terminal half-life in rats was maximal (7.67 days) and clearance reached a minimum of 8.38 ml/day/kg at the highest tested dose level, suggesting a saturable elimination pathway for FG-3019. This PK behavior is consistent with participation of a target-mediated pathway in the elimination of FG-3019. The potential for CTGF to mediate antibody clearance was confirmed in co-administration experiments in which exogenously added rhCTGF rapidly (<3 min) and stoichiometrically eliminated FG-3019 from plasma. The rapidity of this plasma elimination is consistent with the PK of CTGF, which was also very rapidly eliminated from plasma after administration to rats. Together, these data indicate that FG-3019 is subject to target-mediated elimination that contributes to its unusually short half-life and fast clearance.

Monoclonal antibodies targeting soluble antigens with low endogenous circulating concentrations relative to administered antibody (as in the case of FG-3019) generally exhibit dose-independent clearance (20). This is because the interaction of a high concentration of antibody with a low concentration of circulating antigen does not significantly affect the overall rate of antibody clearance. Dose-dependent non-linear elimination kinetics has been observed for antibodies targeting soluble ligands with relatively *high* endogenous concentrations such as IgE (21). The literature suggests that the low circulating concentrations of CTGF result from a low production rate in healthy animals, which increases with disease or after tissue injury (22–24). However, the data presented here indicate that the low plasma concentrations of CTGF normally observed are due to rapid clearance of CTGF, rather than slow production. CTGF is being continuously produced and shed into circulation at a high rate, resulting in rapid clearance of FG-3019 due to target mediated elimination.

Direct measurement of the *in vivo* production rate of CTGF is not feasible. However, the PK data collected for both the antibody and its target, CTGF, enabled construction of a model for computation of various parameters that regulate target and antibody elimination. Although this model represents a simplification of the synthesis and distribution of CTGF and the result of introducing FG-3019 into circulation with its subsequent distribution and elimination, it predicts the PK of CTGF, CTGF-N and FG-3019 reasonably well, as demonstrated by the fit curves in Figs. 1, 2, and 3. Using this model, we can estimate that CTGF is shed into circulation at a constant rate of 0.29 mg/kg/day. In healthy rats, CTGF mRNA expression in tissues is not significantly altered at 24-h after dosing with 100 mg/kg FG-3019, suggesting that the constitutive rate of CTGF production is unaffected by FG-3019 (unpublished results). It remains to be determined which tissues contribute most to this CTGF blood production rate, although cardiac atria have been reported to express high concentrations of CTGF protein (25).

Once CTGF enters the circulation, its clearance appears to be primarily through liver uptake, as demonstrated by the redistribution of [^125^I]-FG-3019 upon co-administration of rhCTGF. Uptake by the liver is also consistent with its rapid elimination, since approximately 100% of a rat’s blood flows through the liver per minute, with about 72.5% per minute flowing through the portal vein (26). Elimination of CTGF differs from that of CTGF-N, which in mice is primarily eliminated through glomerular filtration, consistent with its low molecular weight (2).

CTGF-N is produced from CTGF by proteolytic cleavage in the linker region. Because CTGF is continually being made and shed into circulation, the same is likely true for CTGF-N. The epitope to which FG-3019 binds on CTGF is in the second domain of CTGF-N. Therefore, when FG-3019 is administered and binds to CTGF-N, it increases the apparent molecular weight of CTGF-N above the renal filtration molecular weight cut-off, leading to its accumulation in circulation. The accumulation of other antibody-ligand complexes in the circulation following antibody administration has been modeled successfully (27). For example hepcidin, a small peptide hormone which is normally excreted by renal filtration, accumulates in the circulation in an antibody complex (10). The observation of increased circulating concentrations of CTGF-N upon FG-3019 administration, coupled with the fact that co-administration of rhCTGF-N had no impact on the clearance of FG-3019, while whole CTGF significantly enhanced it, indicates that the motif that mediates liver uptake of CTGF resides in its C-half.

Several cell surface molecules have been reported to interact with CTGF domains 3 or 4 (28). Among them, one of the most likely candidate receptors mediating uptake of CTGF into liver is LRP1. LRP1 is a scavenger receptor that has been reported to bind many ligands (29) including CTGF (30) . In the liver, LRP1 is expressed in both hepatocytes and Kupffer cells (resident macrophages) and it functions to clear plasma protein ligands (29). LRP1 in macrophages also suppresses vascular remodeling by modulating response to TGFβ (31), which may occur at least partially though uptake of CTGF (31). In chondrocytes, LRP1 is thought to mediate CTGF transcytosis (32) . There are also two closely related members of the LRP1 family that are expressed in the adrenal gland, LRP1b (33) and kidney, LRP2/megalin (34,35). LRP2 has been shown to bind CTGF in the kidney, and mediate its excretion (2). Thus, the localization of these three related scavenger receptors could account for the majority of the rapid clearance of [^125^I]-FG-3019 upon co-administration of rhCTGF in the QWBA experiment.

The cell surface expression of LRP1, LRP1b and LRP2 is aided by the endoplasmic reticulum (ER) chaperone RAP (receptor-associated protein, LRPAP1), which binds tightly to these receptors at neutral pH preventing premature ligand binding during folding and transport to the cell surface (29,36). RAP is localized to the ER by a 4 amino acid sequence at its C-terminus, but is secreted in a soluble form when these amino acids are omitted (37). When injected into animals, RAP is rapidly cleared from the circulation via uptake by the liver and kidney (7). Despite its rapid clearance, administration of the soluble form of RAP inhibits plasma clearance of tissue type plasminogen activator and tissue factor pathway inhibitor in rats, presumably by interfering with binding of these factors to LRP1 and related proteins (7,38). Similarly, co-administration of RAP with FG-3019 and rhCTGF resulted in a measurable slowing of the clearance of FG-3019. This observation suggests that at least part of the clearance of CTGF (and CTGF/FG-3019 complexes) from circulation is via binding to one or more of these scavenger receptors. Additional experiments will be necessary to assess the extent of CTGF clearance that is mediated by LRP1 and related scavenger receptors.

Pharmacokinetic modeling of target-mediated drug disposition (TMDD) predicts that the true terminal rate of elimination is independent of the dose of drug administered (13). This can be seen in the parallelism of the terminal FG-3019 disappearance rates in the curves obtained from the kinetic model shown in Fig. 1. However, as can also be seen in Fig. 1, sample collection from animals administered high doses of FG-3019 was terminated too soon to establish the true terminal elimination rates. This failure to collect samples at sufficiently late time points explains the apparent dose-dependence of the experimentally determined half-lives. Another interesting observation from computer modeling is that the affinity of FG-3019 for CTGF appears to be at least 120-fold weaker *in vivo* than predicted by the affinity determined *in vitro* (by radioimmunoassay). This suggests that blood flow or components in blood may weaken the antibody-ligand interaction. As shown in Fig. 4 and previously mentioned, co-administration of rhCTGF rapidly eliminated circulating FG-3019 in proportion to the amount of CTGF administered. When dosed at a 2:1 molar ratio to FG-3019, CTGF caused more than 90% of the FG-3019 in plasma to disappear from circulation in less than 3 min. To our knowledge, the magnitude of rhCTGF’s effect on FG-3019 pharmacokinetics is unprecedented for target-mediated antibody elimination by a soluble ligand. Similar behavior has been described for the effect of avidin on synthetically biotinylated IgG, but this is a special case that is not dependent on ligand recognition through the complementarity determining regions of the antibody (39).

The ability of co-administered CTGF to trigger rapid antibody elimination was also observed for two other anti-CTGF monoclonal antibodies that have different binding epitopes on CTGF from the one recognized by FG-3019 (data not shown). This suggests that any anti-CTGF antibody (or any molecule that binds to CTGF) may be subject to target-mediated elimination. Consequently, any tightly associated complex between CTGF and an endogenous binding partner might also be cleared rapidly once the complex dissociates from the extracellular matrix and enters the circulation.

In conclusion, the unusually rapid clearance and short half-life of FG-3019 results at least in part from target-mediated elimination. The data suggest that CTGF is constitutively produced and shed into circulation at a higher rate than has previously been appreciated. Once in circulation, CTGF appears to be removed upon passage through the liver, and in the process facilitates clearance of anything that is bound to it. It remains to be determined if this mechanism has a physiologic role in the homeostasis of any of the putative CTGF binding partners. However, it clearly has relevance for the administration of any inhibitor of CTGF, which will likely be rapidly cleared and therefore will need to be administered at higher doses and/or more frequently than would be necessary if it were not subject to target-mediated elimination.

## Electronic supplementary material

Below is the link to the electronic supplementary material.

ESM 1 (PDF 331 kb)

## Abbreviations

CTGF: Connective Tissue Growth Factor
CTGF-C: C-terminal half of CTGF
CTGF-N: N-terminal half of CTGF
CTGF-whole: Intact CTGF
Ig: Immunoglobulin
IV: Intravenous
K_d_: Dissociation constant
LLOQ: Lower limit of quantitation
PK: Pharmacokinetics
RAP: Receptor associated protein
rhCTGF: Recombinant human CTGF
SD: Sprague–Dawley
SDS-PAGE: Sodium dodecyl sulfate polyacrylamide gel electrophoresis
