# Supplementary material for: FG-3019, a Human Monoclonal Antibody Recognizing Connective Tissue Growth Factor, is Subject to Target-Mediated Drug Disposition
Source: Pharm Res. 2016 Apr 8;33:1833–49. doi: 10.1007/s11095-016-1918-0 (PMC4942499; doi:10.1007/s11095-016-1918-0)
Supplement: Supplementary file 1 — (PDF 331 kb) [file 11095_2016_1918_MOESM1_ESM.pdf]

Supplement to:

FG-3019, a Human Monoclonal Antibody Recognizing Connective Tissue Growth Factor, is Subject to Target-Mediated Drug Disposition

Mitchell C. Brenner, Wojciech Krzyzanski, James Z. Chou, Pierre E. Signore, Cyra K. Fung,
David Guzman, Dongxia Li, Weihua Zhang, David R. Olsen, Viet-Tam L. Nguyen, Carolyn W. Koo, Mark D. Sternlicht and Kenneth E. Lipson

Received: 25 January 2016 /Accepted: 30 March 2016

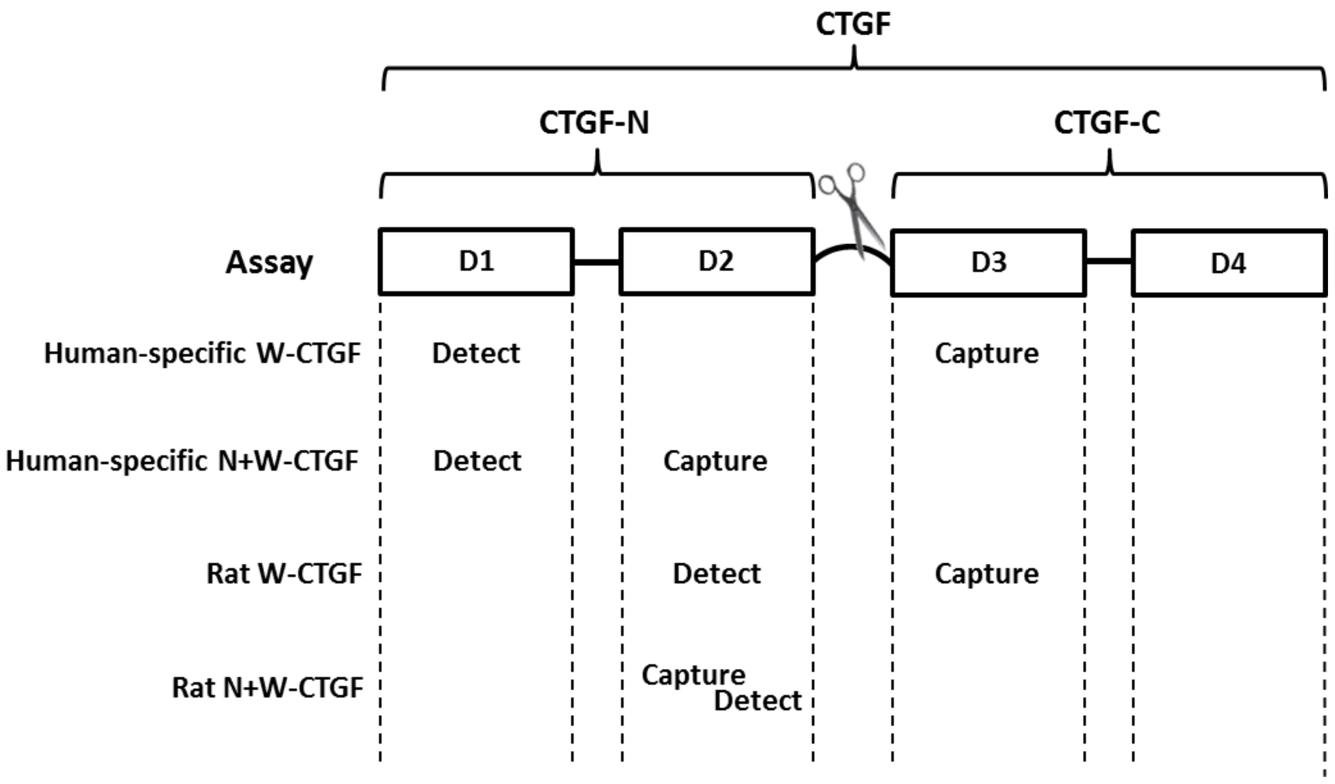

Figure S1. Schematic diagram of the location of the capture and detection antibodies used for ELISA measurements of rat and human CTGF and CTGF-N

Non-Compartmental Pharmacokinetic Parameters

The PK parameters reported here consist of C_{max} (maximum plasma concentration), AUC_{inf} (area under the concentration time curve based on extrapolation to infinity), AUC_{inf}/Dose (dose normalized area under the curve), Cl (clearance, corresponding to the overall rate of elimination of FG-3019 from plasma), V_z (volume of distribution during the terminal phase), V_{ss} (volume of distribution during the steady-state), and $t_{1/2}$ (terminal half-life corresponding to the log-linear slope of the observed terminal phase of the concentration-time profile).

Table S1. Pharmacokinetic Parameters for FG-3019 in Male Sprague-Dawley Rats

	**0.03 mg/Kg**[a]	**0.3 mg/Kg**[a]	**3 mg/Kg**[a]	**10 mg/Kg**[b]	**30 mg/Kg**[b]	**100 mg/Kg**[b]
**C_{max} (μg/mL)**	0.453 (0.072)	5.75 (0.17)	63.3 (7.85)	292 (124)	828 (78)	2665 (375)
**$T_{1/2}$ (days)**	1.36 (0.24)	1.63 (0.45)	1.70 (0.42)	2.62 (0.11)	3.92 (1.55)	7.31 (0.56)
**V_z (mL/Kg)**	178(41.3)	93.6 (22.7)	41.5 (21.8)	64.5 (18.7)	68.1 (28)	94.2 (11.4)
**V_{ss} (mL/Kg)**	118 (20.2)	58.5 (6.1)	48.1 (14.9)	60.6 (20.8)	68.0 (14.8)	87.6 (6.8)
**Cl ($mL*day^{-1}*Kg^{-1}$)**	90.6 (12.9)	40.0 (1.3)	16.0 (5.6)	17 (4.2)	12.2 (1.54)	8.9 (0.8)
**AUC_{inf} ($day*\mu g*mL^{-1}$)**	0.34 (0.05)	7.50 (0.25)	207 (87)	611 (153)	2490 (281)	11300 (1131)
**AUC_{inf}/Dose ($day*\mu g*mL^{-1}*Kg*mg^{-1}$)**	11.2 (1.69)	25.0 (0.82)	69 (29)	61 (15)	83 (9)	113 (11)

a) Values are means of parameters from 3 animals with standard deviations in parentheses. b) Values are averages of parameters from two experiments with 3 animals per experiment. Errors in parentheses are the larger of the standard deviation for the average of two experiments, or the propagated uncertainties for the parameter errors obtained in each experiment.

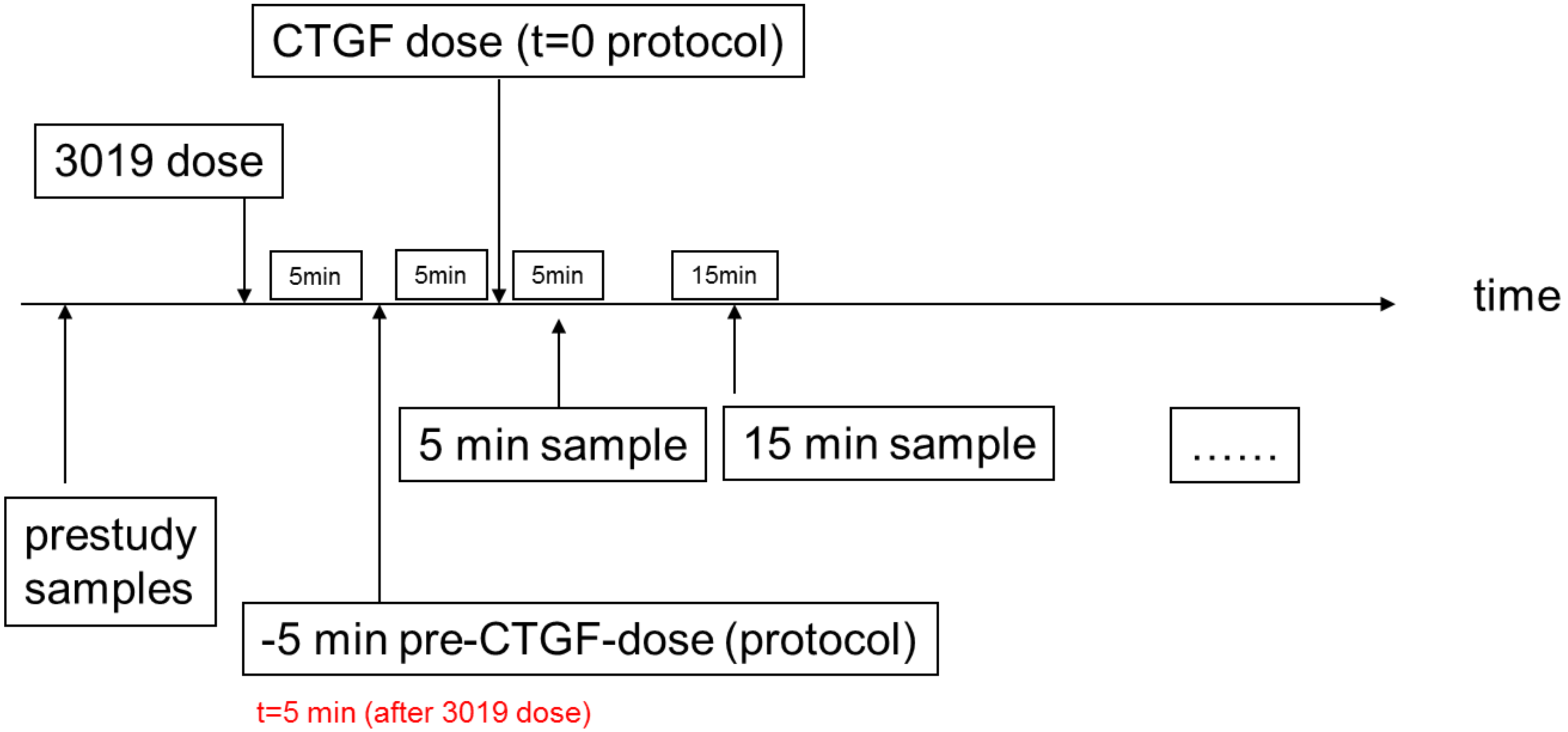

Figure S2. Schematic diagram of the FG-3019 PK experiment in which human CTGF or CTGF-N were co-administered with the mAb in a 1:1 molar ratio.

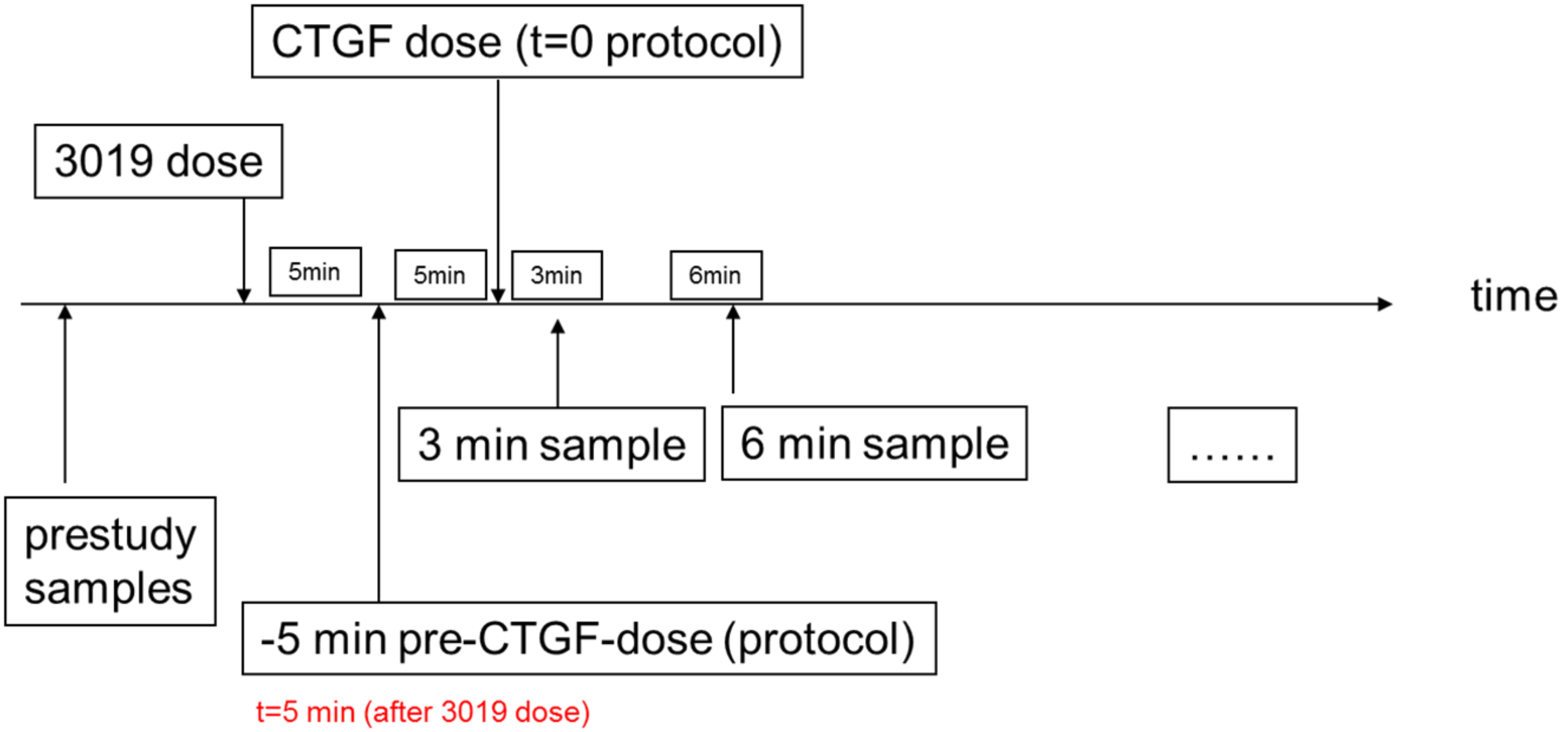

Figure S3. Schematic diagram of the FG-3019 PK experiment in which increasing molar concentrations of human CTGF (0:1, 0.5:1, 1:1, 2:1) were co-administered with the mAb.

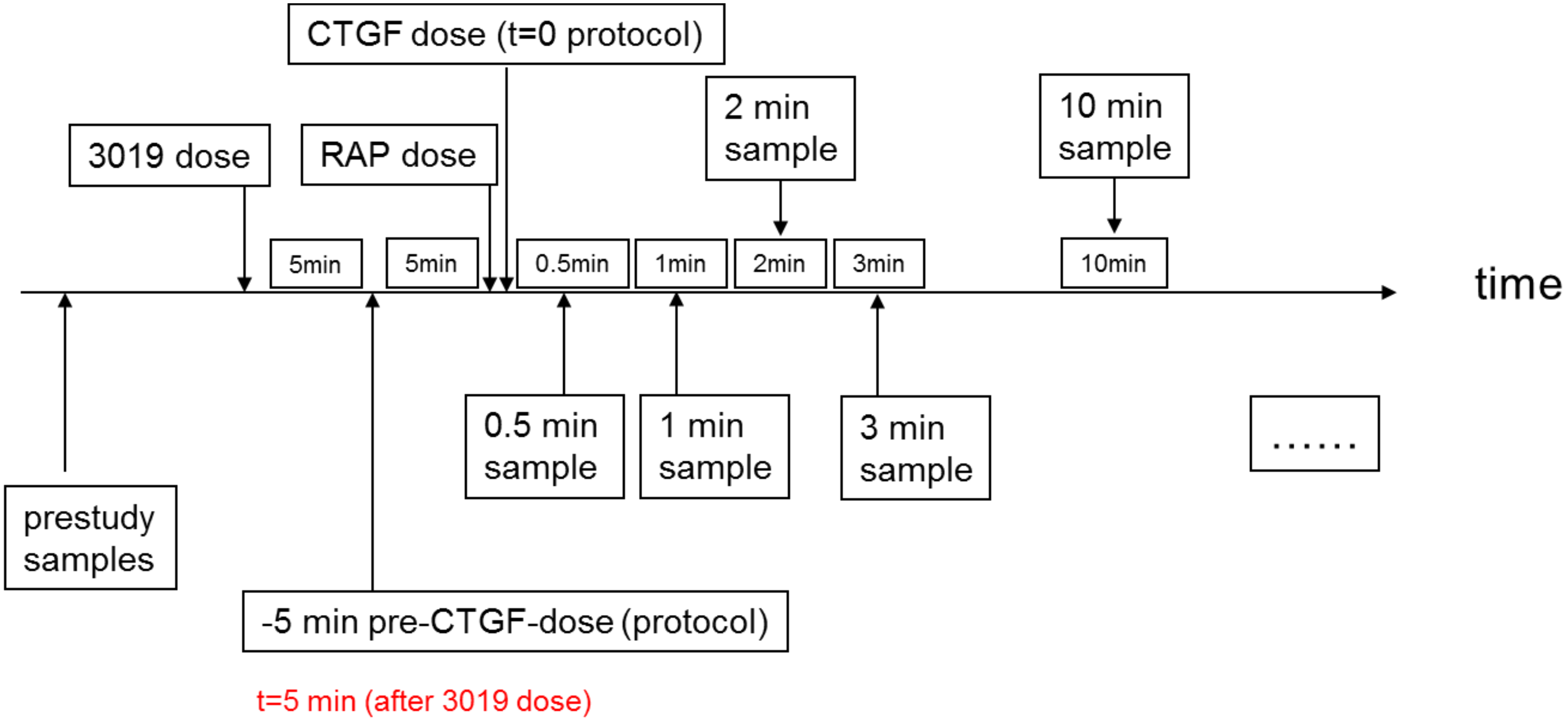

Figure S4. Schematic diagram of the FG-3019 PK experiment in which RAP and a 2-fold molar excess of human CTGF were co-administered with the mAb.

Kinetic Model development

The differential equations corresponding to the kinetic model are as follows:

$$\frac{dW}{dt} = k_W - \frac{CL_W + CL_{dW}}{V} W - k_{onW} \frac{Ab}{V} W + k_{offW} AbW + \frac{CL_{dW}}{V_{WT}} W_T \tag{1}$$

$$\frac{\mathrm{dW_T}}{\mathrm{dt}} = \frac{\mathrm{CL_{dW}}}{\mathrm{V}} \mathrm{W} - \frac{\mathrm{CL_{dW}} + \mathrm{CL_{WT}}}{\mathrm{V_{WT}}} \mathrm{W_T} \tag{2}$$

$$\frac{dN}{dt} = k_N - \frac{CL_N + CL_{dN}}{V} N - k_{onN} \frac{Ab}{V} N + k_{offN} AbN + \frac{CL_{dN}}{V_{NT}} N_T \tag{3}$$

$$\frac{\mathrm{dN_T}}{\mathrm{dt}} = \frac{\mathrm{CL_{dN}}}{\mathrm{V}} \mathrm{N} - \frac{\mathrm{CL_{dN}}}{\mathrm{V_{NT}}} \mathrm{N_T} \tag{4}$$

$$\begin{aligned}\frac{\mathrm{dAb}}{\mathrm{dt}} = &-\frac{\mathrm{CL_{Ab}} + \mathrm{CL_{dAb}}}{\mathrm{V}} \mathrm{Ab} + \frac{\mathrm{CL_{dAb}}}{\mathrm{V_{AbT}}} \mathrm{Ab_T} \\ &- \mathrm{k_{onW}} \frac{\mathrm{Ab}}{\mathrm{V}} \mathrm{W} + \mathrm{k_{offW}} \mathrm{AbW} - \mathrm{k_{onN}} \frac{\mathrm{Ab}}{\mathrm{V}} \mathrm{N} + \mathrm{k_{offN}} \mathrm{AbN}\end{aligned} \tag{5}$$

$$\frac{\mathrm{dAb_T}}{\mathrm{dt}} = \frac{\mathrm{CL_{dAb}}}{\mathrm{V}} \mathrm{Ab} - \frac{\mathrm{CL_{dAb}}}{\mathrm{V_{AbT}}} \mathrm{Ab_T} \tag{6}$$

$$\frac{\mathrm{dAbW}}{\mathrm{dt}} = \mathrm{k_{onW}} \frac{\mathrm{Ab}}{\mathrm{V}} \mathrm{W} - \mathrm{k_{offW}} \mathrm{AbW} - \frac{\mathrm{CL_{AbW}} + \mathrm{CL_{dAbW}}}{\mathrm{V}} \mathrm{AbW} + \frac{\mathrm{CL_{dAbW}}}{\mathrm{V_{AbWT}}} \mathrm{AbW_T} \tag{7}$$

$$\frac{\mathrm{dAbW_T}}{\mathrm{dt}} = \frac{\mathrm{CL_{dAbW}}}{\mathrm{V}} \mathrm{AbW} - \frac{\mathrm{CL_{dAbW}} + \mathrm{CL_{AbWT}}}{\mathrm{V_{AbWT}}} \mathrm{AbW_T} \tag{8}$$

$$\frac{\mathrm{dAbN}}{\mathrm{dt}} = \mathrm{k_{onN}} \frac{\mathrm{Ab}}{\mathrm{V}} \mathrm{N} - \mathrm{k_{offN}} \mathrm{AbN} - \frac{\mathrm{CL_{AbN}} + \mathrm{CL_{dAbN}}}{\mathrm{V}} \mathrm{AbN} + \frac{\mathrm{CL_{dAbN}}}{\mathrm{V_{AbNT}}} \mathrm{AbN_T} \tag{9}$$

$$\frac{\mathrm{dAbN_T}}{\mathrm{dt}} = \frac{\mathrm{CL_{dAbN}}}{\mathrm{V}} \mathrm{AbN} - \frac{\mathrm{CL_{dAbN}}}{\mathrm{V_{AbNT}}} \mathrm{AbN_T} \tag{10}$$

where V denotes the volume of the plasma compartment common for W, N, Ab, AbW, and AbN, and V_{WT}, V_{NT}, V_{AbT}, V_{AbWT}. and V_{AbNT} are the volumes of the tissue compartments W_T, N_T, Ab_T, AbW_T, and AbN_T, respectively. Because of endogenous production, W, $W_{T,}$ N, and N_T have

non-zero baseline values W_0, W_{T0}, N_0, and N_{T0} that can be calculated from the steady-state equations for (1)-(4) resulting in the following relationships:

$$k_W = \left(CL_W + \frac{V}{V_{WT}} CL_{WT} \right) \frac{W_0}{V} \quad (11)$$

$$k_N = CL_N \frac{N_0}{V} \quad (12)$$

$$W_{T0} = \frac{CL_{dW} V_{WT}}{(CL_{dW} + CL_{WT})V} W_0 \quad (13)$$

$$N_{T0} = \frac{V_{NT}}{V} N_0 \quad (14)$$

The following original parameters were replaced by ones that can be compared with experimental values:

$$k_{onW} = \frac{k_{offW}}{K_{DW}} \quad \text{and} \quad k_{onN} = \frac{k_{offN}}{K_{DN}} \quad (15)$$

where K_{DW} and K_{DN} are equilibrium disassociation constants. Also

$$W_0 = C_{W0} V \quad \text{and} \quad N_0 = C_{N0} V \quad (16)$$

where C_{W0} and C_{N0} are W and N baseline plasma concentrations.

Simulations

The estimated parameter values were used to simulate the time courses of the elimination rates for FG-3019 via target mediated and non-target mediated pathways described by the following equations:

$$\text{Target mediated eliminatio n rate} = \frac{CL_{AbWT}}{V_{AbWT}} Ab_{WT} \quad (17)$$

$$\text{Non} - \text{target mediated eliminatio n rate} = \frac{CL_{Ab}}{V} Ab + \frac{CL_{AbW}}{V} Ab_W + \frac{CL_{AbN}}{V} Ab_N \quad (18)$$

Integration of the elimination rates described by Equations (17)-(18) over time yields the amount of FG-3019 (bound and free) eliminated via the target-mediated (A_{Target}) and non-target-mediated

($A_{Non\text{-}target}$) pathways. The integrals were calculated as numerical solutions of the following differential equations:

$$\frac{dA_{Target}}{dt} = \frac{CL_{AbWT}}{V_{AbWT}} Ab_{WT} \tag{19}$$

$$\frac{dA_{Non\text{-}target}}{dt} = \frac{CL_{Ab}}{V} Ab + \frac{CL_{AbW}}{V} Ab_{W} + \frac{CL_{AbN}}{V} Ab_{N} \tag{20}$$

with initial concentrations of all species set to zero. The simulations were performed using the ADAPT 5 program (Biomedical Simulations Resource, 2009).
